# Genome-wide association mapping for resistance to leaf rust, stripe rust and tan spot in wheat reveals potential candidate genes

**DOI:** 10.1007/s00122-018-3086-6

**Published:** 2018-03-27

**Authors:** Philomin Juliana, Ravi P. Singh, Pawan K. Singh, Jesse A. Poland, Gary C. Bergstrom, Julio Huerta-Espino, Sridhar Bhavani, Jose Crossa, Mark E. Sorrells

**Affiliations:** 1 Plant Breeding and Genetics Section, School of Integrative Plant Science, Cornell University, Ithaca, NY 14853 USA; 2 International Maize and Wheat Improvement Center (CIMMYT), Apdo, Postal 6-641, 06600 Mexico, DF, Mexico; 3 Wheat Genetics Resource Center, Department of Plant Pathology and Department of Agronomy, Kansas State University, Manhattan, KS 66506 USA; 4 Plant Pathology and Plant-Microbe Biology Section, School of Integrative Plant Science, Cornell University, Ithaca, NY 14853 USA; 5 Campo Experimental Valle de México INIFAP, 56230 Chapingo, Edo. de México Mexico; 6 CIMMYT, ICRAF house, United Nations Avenue, Gigiri, Village Market, Nairobi, 00621 Kenya

## Abstract

**Key message:**

Genome-wide association mapping in conjunction with population sequencing map and Ensembl plants was used to identify markers/candidate genes linked to leaf rust, stripe rust and tan spot resistance in wheat.

**Abstract:**

Leaf rust (LR), stripe rust (YR) and tan spot (TS) are some of the important foliar diseases in wheat (*Triticum aestivum* L.). To identify candidate resistance genes for these diseases in CIMMYT’s (International Maize and Wheat Improvement Center) International bread wheat screening nurseries, we used genome-wide association studies (GWAS) in conjunction with information from the population sequencing map and Ensembl plants. Wheat entries were genotyped using genotyping-by-sequencing and phenotyped in replicated trials. Using a mixed linear model, we observed that seedling resistance to LR was associated with 12 markers on chromosomes 1DS, 2AS, 2BL, 3B, 4AL, 6AS and 6AL, and seedling resistance to TS was associated with 14 markers on chromosomes 1AS, 2AL, 2BL, 3AS, 3AL, 3B, 6AS and 6AL. Seedling and adult plant resistance (APR) to YR were associated with several markers at the distal end of chromosome 2AS. In addition, YR APR was also associated with markers on chromosomes 2DL, 3B and 7DS. The potential candidate genes for these diseases included several resistance genes, receptor-like serine/threonine-protein kinases and defense-related enzymes. However, extensive LD in wheat that decays at about 5 × 10^7^ bps, poses a huge challenge for delineating candidate gene intervals and candidates should be further mapped, functionally characterized and validated. We also explored a segment on chromosome 2AS associated with multiple disease resistance and identified seventeen disease resistance linked genes. We conclude that identifying candidate genes linked to significant markers in GWAS is feasible in wheat, thus creating opportunities for accelerating molecular breeding.

**Electronic supplementary material:**

The online version of this article (10.1007/s00122-018-3086-6) contains supplementary material, which is available to authorized users.

## Introduction

Leaf rust or brown rust (LR) caused by *Puccinia triticina* Eriks., stripe rust or yellow rust (YR) caused by *Puccinia striiformis* West., and tan spot (TS) caused by *Pyrenophora tritici*-*repentis* (Died.) Shoemaker are some of the important foliar diseases in wheat (*Triticum aestivum* L.). Among these, LR is the most common disease in many wheat-producing areas of the world and can cause substantial yield losses (Marasas et al. 2004), due to reduced kernel number and kernel weight. While the early onset of disease can cause yield losses greater than 50%, losses from 7 to 30% depending on the developmental stage are common (Huerta-Espino et al. 2011). Similarly, YR is a serious disease that is prevalent in the temperate regions and results in yield losses ranging from 10 to 70% (Chen 2005). Besides these rusts, another foliar disease that is globally distributed and economically significant is TS (De Wolf et al. 1998), that can cause yield losses ranging from 18 to 31% under favorable conditions (Bhathal et al. 2003). While fungicides and agronomic practices are available for the management of these diseases, the deployment of resistant cultivars is the most economical and effective strategy.

Plant resistance mechanisms against pathogens are complex. In the first line of defense, conserved molecular signatures of pathogens known as pathogen (or microbe)-associated molecular patterns (PAMPs) are recognized by plant pattern recognition receptors (PRRs) that activate the basal resistance or PAMP-triggered immunity (PTI). Successful pathogens, however, suppress PTI through secreting virulent effector proteins. These effectors activate the second line of defense known as effector-triggered immunity (ETI) mediated by specific disease resistance (*R*) genes (Jones and Dangl 2006). In a typical gene-for-gene interaction between a biotrophic pathogen and a plant, the effectors produced by avirulent (*Avr*) genes in the pathogen are recognized by the corresponding *R*-genes in the plant (Flor 1956) that predominantly encode the nucleotide binding site-leucine rich repeat (NB-LRR) class of proteins (Hammond-Kosack and Jones 1997). Upon this recognition, a hypersensitive response is initiated and leads to localized programmed cell death preventing further colonization by the pathogen. However, selection pressure on the pathogen imposed by large area monoculture and/or long-term deployment of varieties with single *R*-genes leads to strong selection of mutants with virulence. When the frequency of the pathogen population with virulent mutations increases, it results in the breakdown of resistance genes (McDonald and Linde 2002). This has shifted the breeding focus from race-specific/qualitative resistance conditioned by large effect, single *R*-genes to race non-specific/quantitative resistance. Quantitative resistance is generally conditioned by many genes of small effect leading to a preferred mechanism to achieve durability (Johnson 1984). In this type of resistance, the spread of the disease is delayed and is only expressed in adult plants (adult plant resistance, APR) in contrast to *R*-gene resistance that is usually expressed in both seedling and adult plant stages (all stage resistance). To date, more than 74 LR resistance (*Lr*) and 76 YR resistance (*Yr*) genes have been identified and most of them are race-specific except for *Lr34/Yr18/Sr57*, *Lr46/Yr29/Sr58*, *Lr67/Yr46/Sr55*, *Lr68* and *Yr36* (McIntosh et al. 2016). Combinations of *R*-genes with APR genes are expected to provide good levels of durable rust resistance (Kolmer et al. 2009; Ellis et al. 2014).

The interaction of wheat with necrotrophic fungus, *P. tritici repentis* does not follow the gene-for-gene model. This pathogen secretes necrotrophic effectors (also known as host-selective toxins) (Friesen et al. 2008) that interact with a corresponding host sensitivity gene and result in a compatible susceptible interaction. This is referred to as effector-triggered susceptibility (ETS) and the interaction is described as an inverse gene-for-gene model (Friesen et al. 2007). Since, susceptible cultivars could rapidly select for pathogen populations carrying the necrotrophic effectors, breeding efforts focus on eliminating the known susceptibility genes. Six TS resistance genes, *Tsr1/tsn1* (Faris et al. 1996)*, Tsr2/tsn2* (Singh et al. 2006)*, Tsr3/tsn3* (Tadesse et al. 2006a)*, Tsr4/tsn4* (Tadesse et al. 2006b)*, Tsr5/tsn5* (Singh et al. 2008) and *Tsr6/tsc2* (Friesen and Faris 2004) have been identified.

Genomics-assisted breeding for disease resistance typically involves gene identification, isolation, cloning, functional characterization to elucidate the genetic mechanism of resistance, validation and deployment. Resistance genes can be identified by either linkage mapping or genome-wide association studies (GWAS) that are based on linkage disequilibrium (LD) between a marker and the causal polymorphism. GWAS provides a much finer resolution than linkage mapping because it accounts for greater allelic diversity at a given locus and exploits the ancestral recombination events that have occurred in an existing diversity panel at the population level (Yu and Buckler 2006). It has been successfully used to dissect several complex traits in wheat (Breseghello and Sorrells 2006; Crossa et al. 2007; Yu et al. 2011; Juliana et al. 2015). However, several novel quantitative trait loci (QTL) identified in GWAS studies in wheat have not been validated and functionally characterized which have limited their use in breeding programs. Hence, identifying the potential candidate genes linked to significant markers is important as it can provide better insights into results from GWAS. Although this was not possible with the available genetic maps in wheat, the availability of the population sequencing (POPSEQ) reference map (Chapman et al. 2015) that bridges the genetic and physical maps in wheat has made it feasible. The POPSEQ map was developed by whole-genome shotgun sequencing of wheat cultivars, ‘Synthetic W7984’, ‘Opata’ and their recombinant progenies followed by anchoring of the contigs in an ultra-dense genetic map. The POPSEQ data and the chromosome survey sequence assemblies of *T. aestivum* cv. Chinese Spring [International Wheat Genome Sequencing Consortium (IWGSC), 2014] available at Ensembl plants (Bolser et al. 2016) (http://archive.plants.Ensembl.org/Triticum_aestivum/Info/Index) provide an excellent platform for identifying genes linked to the significant markers with known physical positions in the genome. Hence, our objective was to conduct a GWAS for seedling resistance to LR, YR and TS and APR to YR in wheat, followed by exploring the genes linked to the significant markers using Ensembl plants.

## Materials and methods

### Germplasm utilized

The 45th and 46th International Bread Wheat Screening Nursery (IBWSN) entries comprising 333 lines and 313 lines, respectively, were used for this study. The selected bulk breeding scheme was used to develop these lines that were evaluated in cooperating locations globally. Being advanced breeding lines from CIMMYT’s (International Maize and Wheat Improvement Center) bread wheat breeding program, they are expected to have effective and novel resistance genes, which makes them ideal for association mapping.

### Phenotypic evaluations for leaf rust, tan spot and stripe rust

Seedling evaluations for LR and TS were conducted in CIMMYT’s greenhouses at El Batan, Mexico for the 45th IBWSN entries. For LR, freshly collected urediniospores (race MBJ/SP) were suspended in light mineral oil, Soltrol (Phillips 66 Co., Bartlesville, OK, USA) and inoculation was done at the two-leaf stage. The plants were placed in a dew chamber overnight and then transferred to the greenhouse where the minimum, maximum, and average temperatures were 16.1, 30.0 and 20.3 °C, respectively. The 0–4 scale described in Roelfs et al. (1992) was used to evaluate the seedling infection types at 10 days post-inoculation. The scores were linearized to a 0–9 scale as follows:; = 0, 0 = 0, 1 − = 1, 1 = 2, 1 + = 3, 2 − = 4, 2 = 5, 2 + = 6, 3 − = 7, 3 = 8, 3 + = 9 and 4 = 9. For TS, the isolate Ptr1 (Race 1) that produces PtrToxA and PtrToxC (Singh et al. 2009) was used. Inoculum preparation was done as described in Singh et al. (2011) and the concentration was adjusted to 4000 conidia/ml for both seedling and field inoculation. Four seedlings were used to represent each entry and checks Erik, Glenlea, 6B-662 and 6B-365 were planted every 20 rows. Seedling response was evaluated 7 days post inoculation on a 1–5 lesion rating scale developed by Lamari and Bernier (1989). Two replications were scored for LR and six replications were scored for TS.

Seedling and APR to YR were evaluated for the 46th IBWSN entries. While seedling evaluation was conducted in CIMMYT’s greenhouses at El Batan, Mexico, APR evaluations were performed at Toluca, Mexico during the 2011 and 2013 crop seasons, at Quito, Ecuador in 2012 season and at the Kenya Agricultural and Livestock Research Organization, Njoro, during the 2011 main season. For seedling evaluation, inoculum preparation and inoculation were similar to that of LR and the *P. striiformis* race, Mex96.11 was used. The seedlings were incubated in a dew chamber in the dark for 48 h at 7 °C and then transferred to the greenhouse where the minimum, maximum, and average temperatures were 6.3, 30.9 and 17.3 °C, respectively. The YR infection types were recorded at 14 days post-inoculation using a 0–9 scale as described by McNeal et al. (1971). For YR APR evaluation, the lines were sown in 0.7-m long paired rows on top of 30-cm-wide raised beds. The spreaders consisted of a mixture of six susceptible wheat lines derived from an Avocet/Attila cross. The 4-week old spreaders and hills were inoculated three times, at three to 4 days intervals with mixed *Pst* isolates, Mex96.11 and Mex08.13. While Mex96.11 is virulent to *Yr27* and avirulent to *Yr31*, it is the reverse for Mex08.13. Evaluations were conducted at three time points between early and late dough stages. The first evaluation was done when the severity of susceptible check, Avocet reached 80% followed by two more evaluations at weekly intervals. The modified Cobb Scale (Peterson et al. 1948) was used to score rust severity by determining the percentage of infected tissue (0–100%).

All the phenotyping data were transformed using the Box–Cox transformation (Box and Cox 1964).

### Genotyping and linkage disequilibrium analysis

Genome-wide markers were obtained for the lines using genotyping-by-sequencing (GBS) as described by Poland et al. (2012). Markers with missing data greater than 50% and minor allele frequency less than 10% were filtered, which resulted in in 3510 and 8072 markers with known positions for the 45th and 46th IBWSN, respectively. Marker missing data were imputed using the expectation–maximization algorithm implemented in the rrBLUP software package (Endelman 2011). After filtering the lines for missing data greater than 50%, we obtained 267 lines and 305 lines in the 45th and 46th IBWSN, respectively. The pairwise LD between the markers based on their correlations (*R*^2^) was calculated using the ‘R’ statistical program and markers with R2 greater than 0.95 were removed for redundancy.

### Genome-wide association mapping

Genome-wide association mapping employed the mixed linear model (MLM) (Yu et al. 2006) accounting for both population structure and kinship, in TASSEL (Trait Analysis by aSSociation Evolution and Linkage) (Bradbury et al. 2007), version 5.2.24. As population structure can result in spurious associations, it was taken into account by using the first two principal components (Price et al. 2006), calculated in TASSEL using the correlation matrix. Since, there were several sibs in both nurseries, the kinship matrix obtained using the centered identity-by-state method (Endelman and Jannink 2012) was used as a random effect to account for the degree of relatedness between sibs. The MLM was run with the optimum level of compression and the ‘population parameters previously determined’ method (Zhang et al. 2010). To correct for multiple testing, the step up procedure of Benjamini and Hochberg (1995) which controls the false discovery rate was used with a cut-off value of 0.2. We have also used only non-redundant markers to facilitate the reduction of the multiple-testing problem. To find the candidate genes linked to significant markers, the physical starting point of the marker preceded by the chromosome name was taken to Ensembl and a few thousand base pairs were added before and after (e.g. if the position of the marker was 944423 on chromosome 2A, we used 2A: 942423–946423). The number of base pairs added varied for each marker depending on its proximity to the genes, but only the genes that were in the same genetic position were considered. The interval was then explored for predicted genes and annotations that were available from the IWGSC were obtained. For several genes, the IWGSC annotations were not available and so we evaluated orthologous genes in related species with known predicted functions using the comparative genomics tool in Ensembl. The closest species, *Triticum urartu* (A-genome donor) and *Aegilops tauschii* (D-genome donor) were first considered and when orthologs were not available or annotated in them, more distant species including barley (*Hordeum vulgare*), Brachypodium (*Brachypodium distachyon*), rice (*Oryza sativa*), maize (*Zea mays*), foxtail millet (*Setaria italica*), thale cress (*Arabidopsis thaliana)* and banana (*Musa acuminata*) were considered. In some cases, when the genes had a less similar disease resistance ortholog (< 70%) in the annotated genomes of related species in Ensembl, the sequence of the *T. aestivum* gene was taken to NCBI and the nucleotide basic local alignment search tool (BLAST) (http://blast.ncbi.nlm.nih.gov/Blast.cgi) was used where only highly similar sequences (megablast) were considered. This search also included the gene predictions in different species available in GenBank, but not in Ensembl. We also looked at the *T. aestivum* gene transcripts and their domains that were available in Ensembl (using the show transcript table link). We also used Viroblast (https://triticeaetoolbox.org/wheat/viroblast/viroblast.php) in the Triticeae Toolbox website to perform a nucleotide BLAST (BLAST-n) of the significant marker sequences against the GBS markers in Triticeae Toolbox (T3) database. In addition, the JBrowse tool from T3 and GBrowse from URGI (https://urgi.versailles.inra.fr/gb2/gbrowse/wheat_survey_sequence_annotation) were also used to identify other GBS markers and/or markers from the 90K iSelect assay (Wang et al. 2014) that were synonymous to the significant markers in this study.

## Results

### Phenotyping and genotyping data analysis

In the 45th IBWSN, the mean LR seedling score was 7.0 ± 2.1 on a 0–9 scale and the mean TS seedling score was 2.6 ± 0.8 on a 1–5 scale. The correlation of the mean LR seedling score with the mean TS seedling score was very low (− 0.11, respectively). In the 46th IBWSN, the mean YR seedling score was 6.2 ± 2.1 on a 0–9 scale. In contrast, the mean YR severities on a 0–100% severity scale were only 5.5 ± 8.8 (Quito 2012), 6.1 ± 6.6 (Njoro 2011), 2 ± 3.2 (Toluca 2011) and 8.7 ± 6.5 (Toluca 2013), despite high disease pressures leading to 100% severity for the susceptible check.

The percentage of missing data and minor allele frequencies for both the nurseries are shown in Supplementary Fig. 1. We analyzed the relative percentage of markers in each chromosome and observed that chromosome 2B had the highest percentage (~ 12.6%) of markers in both the nurseries followed by chromosomes 3B (~ 11%), 5B (~ 8%), 2A (~ 7.4%) and 7A (~ 7%). Chromosomes 1A, 1B, 6A, 7B, 6B, 4A and 3A had about 5% of the markers each. Chromosomes 5A (~ 3%) and 4B (~ 2.5%) had the lowest percentage of markers in the A and B genomes, respectively. Overall, the D-genome had the lowest number of markers. It ranged from 1.3 to 2.2% on chromosomes 7D, 1D, 6D, 2D and 5D in both nurseries, while, chromosomes 3D and 4D had the least number of markers (less than 1%).

### Linkage disequilibrium and principal component analysis

**Fig. 1:**
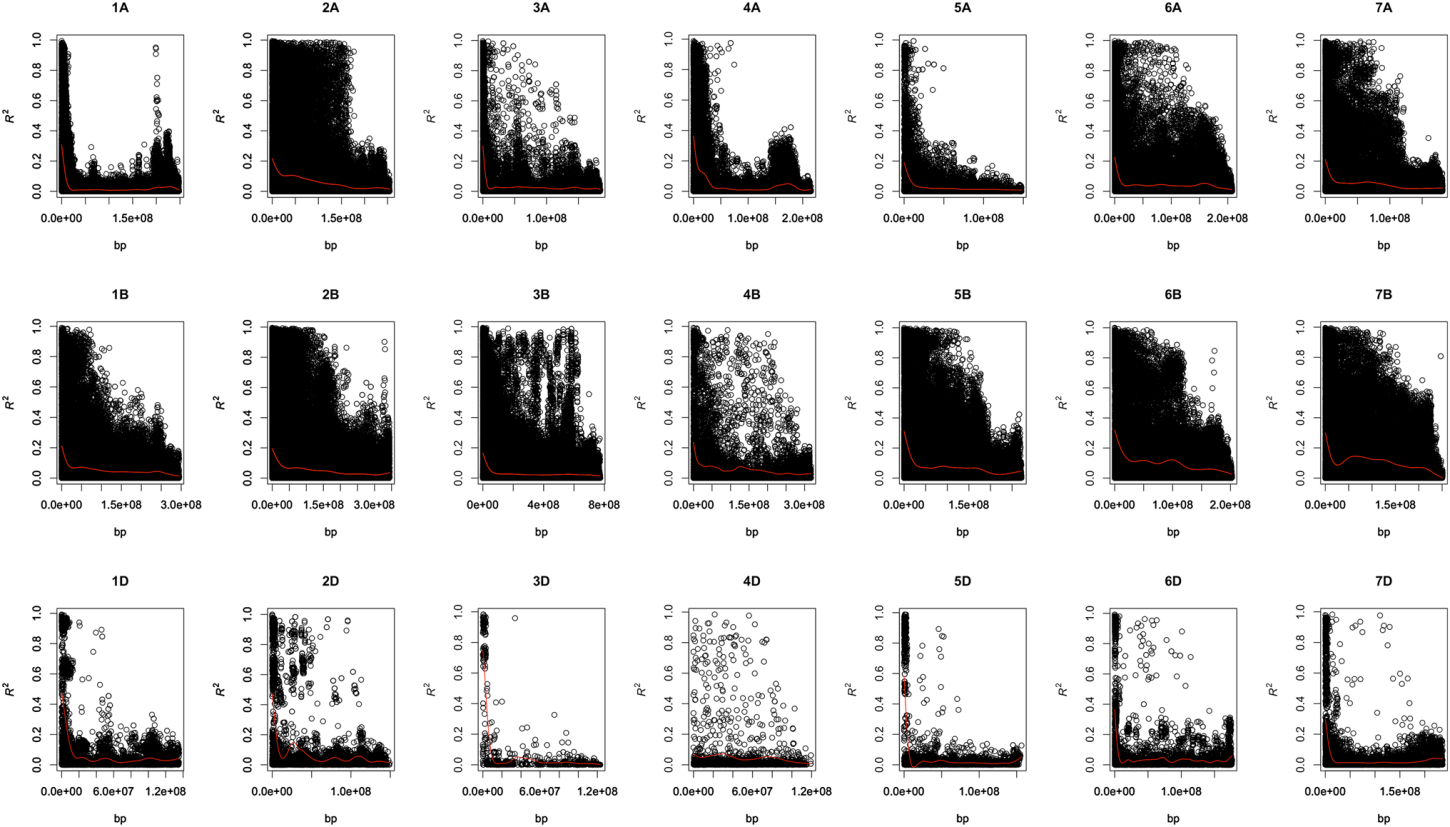
Scatter plot showing the linkage disequilibrium (LD) decay across the chromosomes. The physical distance in base pairs is plotted against the LD estimate (*R*^2^) for pairs of markers in the 46th International Bread Wheat Screening Nursery

Linkage disequilibrium estimated as the allele frequency correlations (*R*^2^) between the GBS markers across the chromosomes was plotted against the physical distance in base pairs (bps). Similar trends of LD decay were observed in both the nurseries. Hence, only the LD decay for the 46th IBWSN is shown in Fig. 1 and that for the 45th IBWSN is shown in Supplementary Fig. 2. The average extent of LD considered as the physical distance taken for the decay of *R*^2^ to a critical value of 0.10 across the genome was approximately 5 × 10^7^ bps.

Principal component analysis revealed that there was moderate population structure in both nurseries. We also identified lines with common parents and observed clear grouping of families. The lines that did not have common parents or had less than three sibs per family were classified as ‘others’. In the 45th IBWSN, the first two principal components explained 9.4 and 7% of the variance, respectively (Supplementary Fig. 3). We observed clear clustering of lines with ‘Kachu’ and ‘Saual’ as parents. Lines with ‘Attila and PBW65’ were closely related to lines with ‘Munal’ in the pedigree. A family with ‘Attila, PBW65, Bobwhite, Neelkant and Catbird’ as parents was clearly different from others and clustered separately. In the 46th IBWSN, the first two principal components explained 10.5 and 6% of the variance, respectively (Supplementary Fig. 4). Lines with ‘Kachu’ in the pedigree formed a separate cluster, similar to the 45th IBWSN. Lines with ‘Mutus’ and ‘Kauz, Minivet, Milan and Baviacora 92’ in the pedigree were very similar. Lines with ‘Weebil 1’, ‘Weebil 1 and Brambling’, ‘Weebil 1 and Kukuna’, ‘Becard and Quaiu’ and ‘Chyak’ as parents, clustered together. Sibs from a cross between ‘Becard and Francolin’ clustered separately.

### Genome-wide association mapping

The markers significantly associated with LR, TS and YR, their chromosomal locations, *p* values, closest *T. aestivum* gene(s), orthologous gene (only the ortholog with the highest identity is reported), the query percent identity (the percentage of the sequence in the *T. aestivum* gene that matches to the ortholog), predicted function and the domains present in the *T. aestivum* gene transcripts are reported (Tables 1, 2, 3). The adjusted *p* values for the markers, *R*^2^ values, synonymous/nearest markers and locations of the *T. aestivum* genes are also reported (Supplementary Tables 1–3). If several genes were in the same genetic position as the significant marker, only the adjacent gene(s) is/are reported. This is because the average LD decay was 5 × 10^7^ base pairs and it is not feasible to report all the genes that lie within this window. Further information on the adjacent genes that lie beyond the marker’s genetic position can be obtained either from Ensembl or from Popseq Ordered Triticum Aestivum Gene Expression (POTAGE), which is a web application integrating POPSEQ map location information with functional annotations and gene expression data (http://130.56.251.241/potage/). Quantile–quantile plots of *p* values comparing the uniform distribution of the expected − log_10_ *p* value to the observed − log_10_ *p* value for different traits showed that the MLM fits the data well, except for a few datasets that had low power to detect significant associations (Supplementary Figs. 5 and 6). Manhattan plots showing the − log_10_ *p* values of the markers in the different datasets from the 45th and 46th IBWSNs are shown in Supplementary Figs. 7 and 8.

**Table 1:** Markers significantly associated with seedling resistance to leaf rust

Marker	Chromosome	Genetic position	*p* value	Adjacent *T. aestivum* gene	Orthologous gene	Identity	Predicted function
S3_6957300	1DS	25.4	1.40E−09	Traes_1DS_3CC12E215	BRADI3G24960^a^	95	Armadillo repeat
				Traes_1DS_A8BD91E4A	F775_28014^b^	100	PAL
S3_1241625	1DS	2.7	1.20E−08	Traes_1DS_3C6EAAFFD	TRIUR3_19829^c^	86	Putative disease resistance protein RGA4
S16_199359368	6AL	118.5	3.80E−07	Traes_6AL_5A3E5FBBD	F775_22846^b^	92	Pentatricopeptide repeat
S16_50275005	6AS	61	8.50E−06	Traes_6AS_EB7270F83	TRIUR3_12413^c^	96	LRR receptor-like STPK
S10_147185899	4AL	29	3.30E−05	Traes_4AL_5EC714CAD	TRIUR3_02349^c^	93	Beta-glucosidase
S8_40178495	3B		6.60E−05	TRAES3BF078500390CFD_g	LOC100835928^a^	85	STPK
S16_197872823	6AL	88.2	7.30E−05	Traes_6AL_C41FC1A58			Lipid transporter
S8_13948258	3B		7.60E−05	TRAES3BF060400070CFD_g	F775_11633^b^	89	E3 ubiquitin-protein ligase SINA-like protein 4
S8_1092429	3B		5.90E−04	TRAES3BF035300120CFD_g	TRIUR3_01154^c^	97	Subtilisin-like protease
S8_667573277	3B		6.40E−04	TRAES3BF068900010CFD_g	F775_05560^b^	96	Endoribonuclease Dicer-3a-like protein
S5_344241063	2BL	153.8	6.50E−04	Traes_2BL_48E8EC589	F775_13446^b^	84	Putative LRR receptor-like STPK
S4_944423	2AS	0	7.80E−04	Traes_2AS_F19BE023F	TRIUR3_09185^c^	92	Putative disease resistance protein RXW24L

*LRR* leucine rich repeat, *PAL* phenylalanine ammonia-lyase, *RGA* resistance gene analog, *SINA* seven in absentia, *STPK* serine/threonine-protein kinase

^a^Gene from *B. distachyon*

^b^Gene from *A. tauschii*

^c^Gene from *T. urartu*

**Table 2:** Markers significantly associated with seedling resistance to tan spot

Marker	Chromosome	Genetic position	*p* value	Adjacent *T. aestivum* gene	Orthologous gene	Identity	Predicted function
S1_3589926	1AS	28	2.40E−05	Traes_1AS_BF353B963	LOC100824961^a^	79	Putative disease resistance protein RPM1
				Traes_1AS_F098402B4	TRIUR3_05134^c^	98	Bowman-Birk type trypsin inhibitor
S7_182028651	3AL	197.4	3.60E−04	Traes_3AL_91749D67D	F775_07165^b^	94	Putative disease resistance protein RGA4
S4_239686345	2AL	113.9	3.60E−04	Traes_2AL_34A3B95BE	GSMUA_Achr11G12630_001^e^	42	Putative disease resistance response protein 206
					LOC101765197^f^	73	Dirigent protein 1-like
				Traes_2AL_97FC5264A	TRIUR3_01787^c^	96	Wall-associated receptor kinase-like
S8_12198705	3B		3.80E−04	TRAES 3BF270500020CFD_g	OS01G0115750^d^	76	STPK
S8_13415415	3B		4.30E−04	TRAES 3BF060400190CFD_g	OS02G0626100^d^	80	Phenylalanine ammonia-lyase
S16_4196814	6AS	13.1	4.90E−04	Traes_6AS_3A682BA20	OS02G0106900^d^	74	STPK
S8_7801088	3B	6.82	5.05E−04	TRAES 3BF060200040CFD_g	BRADI3G16550^a^	87	Hydroxyproline-rich glycoprotein family
				TRAES 3BF060200010CFD_g			ARM repeat superfamily protein
S8_1092429	3B		8.60E−04	TRAES 3BF035300120CFD_g	TRIUR3_01154^c^	97	Subtilisin-like protease
S7_4804454	3AS	12.6	9.00E−04	Traes_3AS_769E90DDD	TRIUR3_11178^c^	90	Putative cysteine-rich receptor-like kinase
S16_191519837	6AL	67.9	1.40E−03	Traes_6AL_4815187D4	LOC100839119^a^	78	LRR receptor-like STPK
S1_2331617	1AS	27.2	2.30E−03	Traes_1AS_AAB89883E1	TRIUR3_12921^c^	99	Disease resistance protein RPM1
				Traes_1AS_459048879	F775_18040^b^	85	Disease resistance protein RPP13
				Traes_1AS_3BE2A2127	F775_01616^b^	76	Putative disease resistance protein
S7_4563676	3AS	12.6	2.30E−03	Traes_3AS_817ECEF75	F775_18635^b^	78	Wall-associated receptor kinase 1
S5_281016023	2BL	97.4	3.30E−03	Traes_2BL_7C2F474DE	TRIUR3_04133^c^	96	Peroxidase
				Traes_2BL_D055B271C	LOC100825682^a^	94	Putative ABC transporter C family member 15
S1_2584791	1AS	28	4.50E−03	Traes_1AS_C8A8A4118	LOC100823561^a^	90	ABC transporter D family member 1
				Traes_1AS_B716E0B0E	LOC100831913^a^	79	Disease resistance protein RPP13
				Traes_1AS_8D33AB43B	TRIUR3_19998^c^	91	ABC transporter D family member 1
				Traes_1AS_C7A8188D1	LOC100830206^a^	80	Disease resistance protein RPM1

*ABC* adenosine triphosphate binding cassette, *ARM* armadillo, *LRR* leucine rich repeat, *RGA* resistance gene analog, *RPM1* resistance to *Pseudomonas syringae* pv. *maculicola* 1, *RPP13* recognition of *Peronospora parasitica* 13, *STPK* serine/threonine-protein kinase

^a^Gene from *B. distachyon*

^b^Gene from *A. tauschii*

^c^Gene from *T. urartu*

^d^Gene from *O. sativa Japonica*

^e^Gene from *M. acuminata*

^f^Gene from *S. italica*

**Table 3:** Markers significantly associated with seedling and adult plant resistance to stripe rust

Dataset	Marker	Chromosome	Genetic position	*p* value	Adjacent *T. aestivum* gene	Orthologous gene	Identity	Predicted function
Njoro 2011	S4_208035	2AS	0	5.50E−04	Traes_2AS_5EB59FFC0	F775_06675^b^	92	PAL
Quito 2012				4.60E−04				
Seedling				2.80E−09				
Toluca 2011				3.90E−08				
Toluca 2013				3.30E−08				
Njoro 2011	S4_508877	2AS	0	6.10E−04	Traes_2AS_6A15EE669	LOC100842644^a^	100	ABC transporter B family member 4-like
Quito 2012				7.70E−08				
Seedling				5.80E−14				
Toluca 2011				3.00E−07				
Toluca 2013				2.40E−10				
Njoro 2011	S4_944423	2AS	0	2.50E−05	Traes_2AS_F19BE023F	TRIUR3_09185^c^	92	Putative disease resistance protein RXW24L
Quito 2012				5.90E−07				
Seedling				7.70E−13				
Toluca 2011				2.40E−07				
Toluca 2013				6.00E−11				
Quito 2012	S4_5007061	2AS	8.9	1.90E−03	Traes_2AS_6BC67DD45	TRIUR3_16539^c^	97	Putative disease resistance RPP13-like protein
Seedling				1.90E−07				
Njoro 2011	S4_5287800	2AS	8.9	6.50E−05	Traes_2AS_A477CDA77	TRIUR3_30356^c^	71	Putative disease resistance RPP13-like protein
Quito 2012				2.60E−07				
Seedling				6.40E−11				
Toluca 2011				1.10E−06				
Toluca 2013				3.10E−08				
Njoro 2011	S4_7117805	2AS	8.9	4.30E−05	Traes_2AS_6CE6AB560	LOC100830175^a^	79	Putative disease resistance protein RGA3
Quito 2012				3.60E−06				
Seedling				3.40E−11				
Toluca 2011				8.20E−06				
Toluca 2013				1.00E−08				
Njoro 2011	S6_132714407	2DL	82.4	1.20E−03	Traes_2DL_4B5D621C1	F775_25858^b^	100	Wall-associated receptor kinase
Quito 2012	S8_17773150	3B		2.00E−05	TRAES3BF050800140CFD_g	TRIUR3_18467^c^	91	Cysteine-rich receptor-like protein kinase
Toluca 2011	S8_566227604	3B		9.30E−04	TRAES3BF027700080CFD_g	F775_04751^b^	94	Putative LRR receptor-like STPK
Toluca 2013	S21_4853558	7DS	3.7	3.00E−04	Traes_7DS_600B0996B	Si017007 m.g^d^	70	Mlo-like protein

*ABC* adenosine triphosphate binding cassette, *LRR* leucine rich repeat, *PAL* phenylalanine ammonia-lyase, *RGA* resistance gene analog, *RPP13* recognition of *Peronospora parasitica* 13, *STPK* serine/threonine-protein kinase

^a^Gene from *B. distachyon*

^b^Gene from *A. tauschii*

^c^Gene from *T. urartu*

^d^Gene from *S. italica*

Seedling resistance to LR was associated with twelve markers (Table 1). Marker S3_6957300 on chromosome 1DS was the most significant marker explaining 19% of the variation. This was followed by markers S3_1241625 (1DS), S16_199359368 (6AL), S16_5027500 (6AS), S10_147185899 (4AL), S8_40178495 (3B), S16_197872823 (6AL), S8_13948258 (3B), S8_1092429 (3B), S8_667573277 (3B), S5_344241063 (2BL) and S4_944423 (2AS). Seedling resistance to TS was associated with 14 markers (Table 2). The most significant marker was S1_3589926 on chromosome 1AS that explained 10% of the variation. Other significant markers include S7_182028651 (3AL), S4_239686345 (2AL), S8_12198705 (3B), S8_13415415 (3B), S16_4196814 (6AS), S8_7801088 (3B), S8_1092429 (3B), S7_4804454 (3AS), S16_191519837 (6AL), S1_2331617 (1AS), S7_4563676 (3AS), S5_281016023 (2BL) and S1_2584791 (1AS).

Seedling resistance to YR was associated with markers: S4_208035, S4_508877, S4_944423, S4_5007061, S4_5287800, S4_7117805 on chromosome 2AS (Table 3). All these markers except S4_5007061 (that was only significant in Quito 2012), were also associated with APR in all the four datasets. The most significant markers for seedling resistance and APR explained 27 and 14% of the variation. The other markers significantly associated with YR APR include S6_132714407 (2DL) in the Njoro 2011 dataset, S8_17773150 (3B) in the Quito 2012, S8_566227604 (3B) in the Toluca 2011 dataset and S21_4853558 (7DS) in the Toluca 2013 dataset.

## Discussion

### Seedling resistance to leaf rust

The two most significant markers for seedling resistance to LR were located on chromosome 1DS. This chromosome has the catalogued LR resistance genes, *Lr21* (Rowland and Kerber 1974), *Lr42* (Cox et al. 1994a) and *Lr60* (Hiebert et al. 2008). Considering the *Lr42* gene, the marker *Xwmc432* that was tightly linked to it (Sun et al. 2010) was at 22.5 cM on the wheat composite map (Somers et al. 2004). As the most significant marker in this study was at 25.4 cM in the POPSEQ map, we believe it to be linked to the *Lr42* gene in that region. Simple sequence repeat markers, *cfd15* and *wmc432* also confirmed the presence of this gene. *Lr42* is a moderately effective race-specific resistance gene that is effective against race MBJ/SP. It originated from an *A. tauschii* introgression line, ‘KS91WGRC11’ (Cox et al. 1994b) and is represented as line Lr42 in CIMMYT pedigrees. This line along with CIMMYT’s spring wheat line ‘Quaiu’ that have the *Lr42* gene (Basnet et al. 2013), were used as parents in some of the crosses and are likely the donors for resistance. The second most significant marker was located at 2.7 cM, about 22.7 cM apart from the other marker. This is close to the location of the *Lr60* gene, that is 8.4 cM distal to the marker *Xbarc149* [13.7 cM in the wheat composite map (Somers et al. 2004)] and approximately 17 cM away from *Lr42*. The cloned NBS-LRR *Lr21* gene (Huang et al. 2003) is also in this location. But the relative position of these genes and the significant marker could not be obtained.

On chromosome 2AS, a marker was significant and the catalogued LR resistance genes in this chromosome are: *Lr17* from bread wheat (Dyck and Kerber 1977), *Lr37* from *Aegilops ventricosum* (Bariana and Mcintosh 1993), *Lr45* from *Secale cereale* (McIntosh et al. 1995) and *Lr65* from a Swiss spelt wheat (Mohler et al. 2012). While *Lr17* and *Lr37* are not effective against the race used, it is unlikely that *Lr45* and *Lr65* are conferring resistance in these lines given their origins. On chromosome 2BL a marker was significant and the catalogued genes in this chromosome are *Lr50* from *T. timopheevii* subsp. *armeniacum* (Brown-Guedira et al. 2003) and *Lr58* from *Ae. triuncialis* (Kuraparthy et al. 2007). However, alien sources with these genes were not used in the crosses.

On chromosome 3B four markers were significant but their genetic positions on the POPSEQ map could not be obtained. The known LR resistance genes on this chromosome include *Lr27* from bread wheat (Singh and McIntosh 1984) and *Lr74* that confers APR (Mcintosh et al. 2014), both of which do not confer seedling resistance to the race used. On chromosome 4AL, a marker was significant. But the catalogued LR resistance genes in this region, *Lr28* from *T. speltoides* (McIntosh et al. 1982) and *Lr30* from the bread wheat cultivar Terenzio (Dyck and Kerber 1981) are unlikely to be present in this nursery as sources with these genes were not used as parents.

On chromosome 6A, two markers were significant on the long arm and one on the short arm. The known LR resistance genes on this chromosome are *Lr56* from *Ae. sharonensis* (Marais et al. 2006), *Lr62* from *Ae. neglecta* (Marais et al. 2009) and *Lr64* from *T. dicoccoides* (Mcintosh et al. 2009), all of which are located in the long arm. However, it is unlikely that any of these genes are conferring resistance in these lines, given that they were alien introgressions and were not used as parents.

Among the genes adjacent to the significant markers (Table 1), some of them could be potential candidate genes for LR resistance, although they must be validated. This included disease resistance proteins, resistance gene analog 4 (RGA4) and RXW24L. The RGAs are those with sequences having homology to the conserved domains of R-genes like the NBS-LRR, P-loop and serine/threonine-protein kinase (STPK) (Hammond-Kosack and Jones 1997). The disease resistance protein, RXW24L is a NBS-LRR gene with a P-loop, a LRR domain and a NB-ARC (nucleotide binding-APAF-1 (apoptotic protease-activating factor-1), R proteins and CED-4 (*Caenorhabditis elegans* death-4 protein) domain. In addition to the resistance genes, several STPK receptors that belong to receptor-like kinases (RLKs) were identified as potential candidates. Few LRR receptor-like STPKs are known to be involved in pathogen defense which include the *Xa21* gene that confers resistance against bacterial blight in rice (Song et al. 1995) and flagellin-sensitive-2 gene in *Arabidopsis* that binds bacterial flagellin (Gómez-Gómez and Boller 2000).

Repeats belonging to the armadillo (ARM) family and the pentatricopeptide family were potential candidates. ARM repeats were initially identified in the *Drosophila* segment polarity gene, armadillo (Nusslein-Volhard and Wieschaus 1980) and are a class of helical repeat proteins involved in protein interactions. The largest class of ARM repeats in *Arabidopsis* contain the U-box domain and a U-box/ARM protein encoded by the rice Spotted leaf1 gene was suggested to be involved in the basal defense signaling against rice blast (Zeng et al. 2004). Pentatricopeptide repeat-containing proteins are ribonucleic acid (RNA)-binding proteins known to play important roles in post-transcriptional processes within the mitochondria and chloroplasts (Delannoy et al. 2007). While they play several physiological roles, some of them are also known to be involved in defense against necrotrophic fungi (Laluk et al. 2011) and diverse pathogens (Park et al. 2014).

Several genes encoding enzymes like beta-glucosidase, E3 ubiquitin-protein ligases, endoribonuclease Dicer, phenylalanine ammonia-lyase (PAL) and subtilisin-like protease were also identified as potential candidates. Beta-glucosidases belong to the family 1 glycoside hydrolases that are known to activate phytoanticipins and serve as triggers of chemical defense in plants against pathogens (Morant et al. 2008). E3 ubiquitin-protein ligases with specific domains are known to be involved in plant defense (Yang et al. 2006; Craig et al. 2009; Dielen et al. 2010). A SINA ligase, SINA3 was recently found to be involved in defense signaling in tomato, suggesting a negative role in plant defense response (Miao et al. 2016). Endoribonuclease Dicer-like proteins are known to regulate plant immunity against an array of pathogens including fungi via the small RNAs processed by them (Gupta et al. 2012; Li et al. 2014; Weiberg et al. 2014). Phenylalanine ammonia lyase (EC 4.3.1.24) is a key enzyme in the phenylpropanoid pathway of higher plants involved in the production of several compounds like lignins, coumarins and flavonoids that are related to plant defense (La Camera et al. 2004). Several studies have reported the induction of the PAL gene in response to fungal elicitors and its association with enhanced fungal defense (Pellegrini et al. 1994; Shadle et al. 2003; Tonnessen et al. 2015). Interestingly, the wheat PAL gene had highly similar orthologs in several other plants indicating that it is conserved across species as observed by Rawal et al. (2013). Subtilisin-like proteases are serine proteases and some of them are known to activate defense related genes (Jordá and Vera 2000; Pearce et al. 2010).

In addition to the disease resistance genes, STPKs and enzymes, a gene encoding a lipid transporter was also a potential candidate. Lipid transport proteins transfer phospholipids between membranes and one of them has been classified as a PR protein family member (PR-14) (van Loon and van Strien 1999). While they play diverse roles, some of them are also known to be involved in systemic resistance signaling (Maldonado et al. 2002) and inhibition of bacterial and fungal pathogens (Regente et al. 2005; Sarowar et al. 2009).

### Seedling resistance to tan spot

For seedling resistance to TS, the most significant marker and two other significant markers were located on chromosome 1AS (27.2 cm and 28 cM), where the catalogued gene is *Tsc1* (Effertz et al. 2002). Marker *Xgwm136* that was 4.7 cM distal to *Tsc1* was at 11 cM in a consensus map (Yu et al. 2014). But it was not possible to determine if the significant markers are linked to this gene. Chromosomes 2AL and 2BL had a significant marker, but no TS resistance gene has been reported in these chromosomes.

On chromosome 3AS, two significant markers were located in the same genetic position (12.6 cM). *Tsr4*, the catalogued gene in this chromosome was 14.9 cM away from the marker *Xgwm2* (Tadesse et al. 2010). *Xgwm2* was at 37 cM on the wheat composite map (Somers et al. 2004) which puts *Tsr4* at about 52 cM. Hence, the significant markers in this study are unlikely to be in the location of the *Tsr4* gene. On the long arm of chromosome 3A, a marker was significant, but no resistance gene has been reported in that location. On chromosome 3B, four markers were significant, but the position of only one marker could be obtained on the POPSEQ map. The known genes on chromosome 3BL are *Tsr2/tsn2* that confers resistance to the necrosis induced by a race 3 isolate (Singh et al. 2006) and *Tsr5/tsn5* that confers resistance to the necrosis induced by a race 5 isolate (Singh et al. 2008). Faris and Friesen (2005) also identified a race non-specific QTL (QTs.fcu-3BL) on chromosome 3BL. While, the marker whose position was known (6.8 cM) was not in the position of any of the known genes or QTL, it was not possible to determine if the other markers coincide with them. Finally, three markers on chromosome 6A were significant and no TS resistance gene has been identified in this chromosome.

Among the genes adjacent to the markers significantly associated with TS (Table 2), were genes encoding disease resistance proteins, RGA4 (discussed earlier), RPM1 (resistance to *Pseudomonas syringae* pv *maculicola* 1), disease resistance response protein 206 and RPP13 (recognition of *Peronospora parasitica* 13). RPM1 is a coiled coil-NBS-LRR disease resistance protein that functions at the plasma membrane by interacting with another plasma membrane localized protein called RPM1-interacting protein 4 and mediates ETI to *P. syringae* (Debener et al. 1991; Mackey et al. 2002). The cloned wheat leaf rust resistance gene, *Lr10* has been reported to be similar to the RPM1 gene (Feuillet et al. 2003). The disease resistance response protein 206 is known to be involved in non-host disease resistance response (Wang et al. 1999; Wang and Fristensky 2001; Choi et al. 2004). It is related to the dirigent protein that is suggested to play a role in conifer defense by lignan and lignin formation (Ralph et al. 2006). RPP13 is a leucine zipper NBS-LRR gene from *Arabidopsis* conferring resistance to several different isolates of *Perenospora parasitica* causing downy mildew (Bittner-Eddy et al. 2000; Bittner-Eddy and Beynon 2001).

In addition to the disease resistance genes, receptor-like STPKs, enzymes like PAL and subtilisin, ARM repeat protein (all of which have been discussed earlier), wall-associated receptor kinases, cysteine-rich receptors, adenosine triphosphate (ATP)-binding cassette (ABC) transporters ABCC15 and ABCD1, peroxidase, Bowman–Birk trypsin inhibitor and hydroxyproline-rich glycoproteins were also identified as potential candidates. Wall-associated receptor kinases are tightly bound to pectin in the cell wall and mediate signals between the plasma membrane and the cell wall (Decreux and Messiaen 2005). WAK1, well-studied among these receptors is a PR protein that is induced by pathogen infection and salicylic acid (He et al. 1998). Cysteine rich receptors are characterized by cysteine residues and repeats of the domain of unknown function 26 on the extracellular domain (Chen 2001). They are known to be induced by pathogen infection and regulate basal plant defense (Ederli et al. 2011; Yeh et al. 2015).

The ABC transporters have two hydrophobic transmembrane domains that form the pathway through which substrates like sugars, amino acids, oligopeptides, inorganic ions, polysaccharides, proteins etc. cross the cell membrane and two nucleotide-binding domains that are located at the cytoplasmic side bind ATP and facilitate the transport process (Higgins 1992). Plant ABC transporters are classified into several sub-families (ABCA–ABCH) and play diverse roles (Verrier et al. 2008). The functions of both the ABCC15 and ABCD1 transporters in plants are unknown.

The enzyme peroxidase (POX, EC 1.11.1.7) is an important component of PTI and its activity leads to the production of reactive oxygen species in response to pathogen attack (Daudi et al. 2012; Mammarella et al. 2015). Increase in POX activity in response to fungal infection has been reported in several studies (Seevers and Daly 1970; Thorpe and Hall 1984; Southerton and Deverall 1990). Bowman–Birk type trypsin inhibitor is a serine protease inhibitor that is known to exhibit antifungal activity (Wissenschafts et al. 2000; Qu et al. 2003; Kuhar et al. 2013). Hydroxyproline-rich glycoproteins are integral components of the primary cell wall of plants that accumulate in defense response to various pathogens (Showalter et al. 1985; Shailasree et al. 2004).

### Seedling and adult plant resistance to stripe rust

Both seedling resistance and APR to YR were associated with markers on chromosome 2AS, indicating that they are linked to an all-stage resistance gene, that might be *Yr17* or a closely linked gene. The *Yr17* gene is located between 0 and 4 cM in the wheat composite map (Somers et al. 2004) which is also the approximate location of our markers (0, 8.9 cM). The gene, *Yr17* was introgressed into the French wheat cultivar ‘VPM-1’ as a translocation segment from the D-genome of *Ae. ventricosa.* Lines with Kachu, Milan and Mutus are expected to have the *Yr17* gene and they were used as parents for several crosses. The *Ventriup* marker that amplifies a region in the 2NS translocation also confirmed the presence of this translocation.

In addition to markers on chromosome 2AS, a marker was significant on chromosome 2DL, two markers on chromosome 3B and one marker on chromosome 7DS. On chromosome 2DL, three genes: *Yr37* from *Ae. kotschyi* (Marais et al. 2005), *Yr54* from the common spring wheat line Quaiu (Basnet et al. 2013) and *Yr55* (Mcintosh et al. 2014) have been catalogued. It is unlikely that the gene linked to the significant marker is *Yr37* because *Ae. kotschyi* was not used in the crosses. The relative position of the *Yr55* gene to the marker could not be obtained. Considering the *Yr54* gene, it is unlikely that this marker is linked to it, although it is present in ‘Quaiu’ that was used as a parent in some of the crosses. This is because *Xgwm301*, the marker linked to *Yr54* was at 107 cM in the wheat composite map (Somers et al. 2004) and the significant marker is at 82.4 cM. On chromosome 3B, two markers were significant but their positions could not be obtained. The catalogued rust resistance genes in this chromosome are *Yr4* from common wheat (Bansal et al. 2009)*, Yr30/Sr2* that occurs in a high frequency in CIMMYT germplasm (Singh et al. 2005)*, Yr57* (Randhawa et al. 2015) and *Yr58* (Chhetri et al. 2016). On chromosome 7DS, a marker at 3.7 cM was significant and *Yr18/Lr34* is the catalogued gene in this chromosome which is present in a significant frequency in the CIMMYT germplasm. But the position of a gene-specific marker, cssfr5 for the *Lr34* gene (Lagudah et al. 2009) at 49.3 cM in the consensus map (Yu et al. 2014) indicates that it is not the gene linked to the significant marker.

The genes adjacent to the markers significantly associated with YR seedling resistance include PAL, ABCB4, disease resistance protein RXW24L, disease resistance RPP13-like protein and disease resistance protein RGA3 all of which have been discussed earlier, except ABCB4. The ABCB sub-family (also known as multi-drug resistance proteins) that includes ABCB4 is known to be involved in auxin transport (Noh et al. 2001; Cho et al. 2012). For YR APR, in addition to these, genes encoding wall-associated receptor kinase, cysteine-rich receptor like protein kinase, LRR receptor-like STPK and Mlo-like protein were also potential candidates. All the candidates except for the Mlo-like protein have been discussed earlier. The Mlo locus in barley has recessive mutations that confer broad spectrum resistance to all known isolates of the powdery mildew fungus (*Blumeria graminis* f. sp. *hordei*) (Jørgensen 1992) and could be a potential candidate.

### A segment in the distal end of chromosome 2AS is rich in disease resistance genes

Marker S4_944423 on chromosome 2AS, was associated with seedling resistance to LR, YR and also APR to YR. In addition, several markers in this chromosomal region (0, 8.9 cM) were significantly associated with both seedling and APR to YR and also APR to TS (unpublished results). This is interesting because the ‘2NS’ translocation segment from *A. ventricosa* on the distal end of chromosome 2AS has been previously reported to carry resistance to many diseases: strawbreaker foot rot (eyespot) caused by *Pseudocercosporella herpotrichoides* (*Pch1*) (Doussinault et al. 1983), YR (*Yr17*), stem rust caused by *P. graminis* (*Sr38*), LR (*Lr37*) (Bariana and Mcintosh 1993), cereal cyst caused by *Heterodera avenae* (*Cre5*) (Jahier et al. 2001), root knot caused by *Meloidogyne* spp. (*Rkn3*) (Williamson et al. 2013) and blast caused by *Magnaporthe oryzae* (Cruz et al. 2016). So, we further explored the 2AS chromosomal region and looked at all the genes in the interval from 0 to 7,123,325 bps where the significant markers were located.

There were 228 genes in this region, among which seventeen had disease resistance orthologs and NB-ARC, LRR and/or P-loop containing nucleoside triphosphate hydrolase domains in their transcripts. This included seven genes with disease resistance RPP13-like protein orthologs, four genes with disease resistance protein RGA3 orthologs, two genes with disease resistance protein RGA2 orthologs, two genes with disease resistance protein RPM1 orthologs, one gene with disease resistance protein RXW24L ortholog and one gene with disease resistance protein ortholog (Supplementary Table 4). Among the other genes, those with defensin, PAL and ABCG transporter family member orthologs are interesting as they are also known to be involved in disease resistance. Plant defensins are cysteine-rich peptides involved in plant innate immunity that are generally active against a broad spectrum of fungal pathogens and other microbes (Broekaert et al. 1995; de Carvalho and Gomes 2009). While the PAL gene has been discussed earlier, Tonnessen et al. (2015) reported a rice PAL gene (*OsPAL4*) that was associated with broad spectrum disease resistance. Finally, the ABCG transporter family (also pleiotropic drug resistance family) members are also known to be involved in plant defense (Stukkens et al. 2005; Stein et al. 2006; Krattinger et al. 2009). We hypothesize that either combinations of *R*-genes or genes that confer broad spectrum resistance are responsible for the multiple disease resistance associated with lines carrying this segment. However, not all the genes in this chromosomal segment might be effective as races MBJ/SP and MCJ/SP are virulent to *Lr37* gene and the Ug99 group of races in Kenya are virulent to *Sr38* gene, both of which are linked to the *Yr17* gene. Among the 228 genes, orthologs were found only for 146 genes. Considering only the orthologs of highest similarity, the species with the most number of genes orthologous to the *T. aestivum* genes in this chromosomal segment was *T. urartu* followed by *A. tauschii*, *M. acuminata*, *B. distachyon*, *O. sativa Japonica* and *Z. mays* (Supplementary Fig. 9).

In conclusion, we have identified several markers and potential candidate genes associated with seedling resistance to LR, YR, and TS and also APR to YR. However, these results should be taken with caution for two reasons: (1) our ability to identify potential candidate genes is limited by the resolution of the GBS markers and the current reference wheat genome assembly. (2) Given the very high LD in wheat, there could be several hundreds of genes in the location of a significant marker and it is not possible to identify the causal gene with just GWAS. Analyzing the significance and the LD of markers around a significant marker will help to delineate the most likely interval for the causal gene. However, extensive LD in wheat that decays at about 5 × 10^7^ bps, poses a huge challenge for delineating candidate gene intervals. The genes in that interval must be narrowed down using fine mapping and the final candidates must be functionally characterized using gene editing, gene silencing or targeting induced local lesions in genomes (TILLING) populations etc. Nevertheless, GWAS is the first step to identify candidate genes at the population level and can provide valuable information on the genetic architecture of the traits.

Our results support previous findings that plant defense mechanisms against pathogens are multifaceted and complex. While ETI mediated by NBS-LRR genes plays an important role, the defense response genes that govern basal resistance and PTI should not be overlooked. However, some resistance genes that are present in a significant frequency in CIMMYT germplasm were not identified in this study. This might be due to several reasons: (1) the limited phenotypic variability in these advanced breeding lines that were selected for rust resistance, (2) the high frequency of these genes in the lines, (3) the exclusion of several GBS markers (that could be potentially associated), because their positions were not available in the POPSEQ map. We also observed that several genes were associated with resistance in the same genetic position. In this case, the physical map can provide better insight into those genes. The disease resistance gene rich segment on chromosome 2AS is very promising and should be explored further for use in breeding. We conclude that identifying candidate genes linked to significant markers in GWAS is feasible in hexaploid wheat using GBS markers, POPSEQ map and Ensembl plants, thus creating opportunities for accelerating gene cloning and molecular breeding in wheat.

#### **Author contribution statement**

PJ drafted the manuscript and performed the research. MS, RPS and PKS planned the study, supervised the analysis and revised the manuscript. JH and SB generated the phenotyping data. JP performed the genotyping. GCB and JC supervised the analysis and critically reviewed the manuscript.

## Electronic supplementary material

Below is the link to the electronic supplementary material.

Supplementary material 1 (DOCX 63 kb)

Supplementary material 2 (TIFF 2189 kb) Supplementary Fig. 1: Distribution of missing data and minor allele frequency of markers in the 45th and 46th International Bread Wheat Screening Nursery entries

Supplementary material 3 (TIFF 52505 kb) Supplementary Fig. 2: Scatter plot showing the linkage disequilibrium (LD) decay across the chromosomes. The physical distance in base pairs is plotted against the LD estimate (R2) for pairs of markers in the 45th International Bread Wheat Screening Nursery

Supplementary material 4 (TIFF 6252 kb) Supplementary Fig. 3: Principal component analysis and clustering of families in the 45th International Bread Wheat Screening Nursery

Supplementary material 5 (TIFF 6252 kb) Supplementary Fig. 4: Principal component analysis and clustering of families in the 46th International Bread Wheat Screening Nursery

Supplementary material 6 (TIFF 1955 kb) Supplementary Fig. 5: Quantile–quantile plots of *p* values comparing the uniform distribution of the expected –log_10_ *p* value to the observed –log_10_ *p* value for leaf rust and tan spot in the 45th International Bread Wheat Screening Nursery

Supplementary material 7 (TIFF 9377 kb) Supplementary Fig. 6: Quantile–quantile plots of *p* values comparing the uniform distribution of the expected –log_10_ *p* value to the observed –log_10_ *p* value for stripe rust seeding and adult plant resistance in the 46th International Bread Wheat Screening Nursery

Supplementary material 8 (TIFF 4377 kb) Supplementary Fig. 7: Manhattan plot showing –log_10_ *p* values of the markers for seedling resistance to leaf rust and tan spot in the 45th IBWSN

Supplementary material 9 (TIFF 5471 kb) Supplementary Fig. 8: Manhattan plot showing –log_10_ *p* values of the markers for seedling and adult plant resistance to stripe rust in the 46th IBWSN

Supplementary material 10 (TIFF 5002 kb) Supplementary Fig. 9: Pie chart showing the species with orthologs of highest similarity to the *T. aestivum* genes in a segment on chromosome 2AS

## Abbreviations

ABC: Adenosine triphosphate binding cassette
APR: Adult plant resistance
ARM: Armadillo
BLAST: Basic Local Alignment Search Tool
CIMMYT: International Maize and Wheat Improvement Center
ETS: Effector-triggered susceptibility
ETI: Effector-triggered immunity
GBS: Genotyping-by-sequencing
GWAS: Genome-wide association studies
IBWSN: International Bread Wheat Screening Nursery
IWGSC: International Wheat Genome Sequencing Consortium
LD: Linkage disequilibrium
LR: Leaf rust
MLM: Mixed linear model
RNA: Ribonucleic acid
NB-ARC: Nucleotide binding-APAF-1 (apoptotic protease-activating factor-1): R proteins and CED-4 (*Caenorhabditis elegans* death-4 protein)
NBS-LRR: Nucleotide binding site-leucine rich repeat
PAL: Phenylalanine ammonia-lyase
PAMPs: Pathogen-associated molecular patterns
POPSEQ: Population sequencing
POTAGE: Popseq Ordered Triticum Aestivum Gene Expression
POX: Peroxidase
PR: Pathogenesis-related
PRRs: Pattern recognition receptors
PTI: Pathogen-associated molecular pattern-triggered immunity
QTL: Quantitative trait loci
RGA: Resistance gene analog
RLK: Receptor-like kinase
RPM1: Resistance to *Pseudomonas syringae* pv. *maculicola* 1
RPP13: Recognition of *Peronospora parasitica* 13
SINA: Seven in absentia
STPK: Serine/threonine-protein kinase
TASSEL: Trait Analysis by aSSociation Evolution and Linkage
TILLING: Targeting induced local lesions in genomes
TS: Tan spot
YR: Stripe rust
