# Supplementary material for: Genome-wide association mapping for resistance to leaf rust, stripe rust and tan spot in wheat reveals potential candidate genes
Source: Theor Appl Genet. 2018 Mar 27;131(7):1405–22. doi: 10.1007/s00122-018-3086-6 (PMC6004277; doi:10.1007/s00122-018-3086-6)
Supplement: Supplementary file 1 — Supplementary material 1 (DOCX 63 kb) [file 122_2018_3086_MOESM1_ESM.docx]

**Supplementary Table 1: Markers significantly associated with seedling resistance to leaf rust**

| Marker | FDR-adjusted p-value | R^2^ | Synonymous/nearest marker | *T. aestivum* gene | Gene location | Domain(s) in *T. aestivum* gene transcripts |
| --- | --- | --- | --- | --- | --- | --- |
| S3_6957300 | 4.91E-06 | 0.19 | - | Traes_1DS_3CC12E215 | 6,962,963-6,970,722 | Armadillo -type fold |
|  |  |  |  | Traes_1DS_A8BD91E4A | 7,031,869-7,045,046 | PAL |
| S3_1241625 | 2.11E-05 | 0.18 | - | Traes_1DS_3C6EAAFFD | 1,236,116-1,237,549 | NB-ARC, P-loop containing nucleoside triphosphate hydrolase |
| S16_199359368 | 3.33E-04 | 0.17 | gbsHWWAMP20056 | Traes_6AL_5A3E5FBBD | 199,359,901-199,366,693 | Pentatricopeptide repeat |
| S16_50275005 | 2.71E-03 | 0.15 | Excalibur_c31593_851 | Traes_6AS_EB7270F83 | 50,272,280-50,275,549 | LRR |
| S10_147185899 | 7.24E-03 | 0.15 | gbsCNLmaster1072, | Traes_4AL_5EC714CAD | 147,182,703-147,186,548 | Glycoside hydrolase, family 1 |
| S8_40178495 | 1.05E-02 | 0.14 | gbsCNLmaster7314, | TRAES3BF078500390CFD_g | 40,077,031-40,080,038 |  |
| S16_197872823 | 1.08E-02 | 0.14 | gbsCNLmaster45486, Ra_c9608_183 | Traes_6AL_C41FC1A58 | 197,866,768-197,875,345 |  |
| S8_13948258 | 1.08E-02 | 0.14 | gbsCNLmaster37632 | TRAES3BF060400070CFD_g | 13,944,796-13,952,647 |  |
| S8_1092429 | 6.09E-02 | 0.12 | gbsCNLmaster44982 | TRAES3BF035300120CFD_g | 1,087,451-1,090,526 |  |
| S8_667573277 | 6.42E-02 | 0.12 | - | TRAES3BF068900010CFD_g | 667,564,781-667,574,259 |  |
| S5_344241063 | 6.44E-02 | 0.12 | gbsCNLmaster1957 | Traes_2BL_48E8EC589 | 344,321,397-344,325,034 | Concanavalin A-like lectin/glucanase domain, LRR, STPK |
| S4_944423 | 7.18E-02 | 0.11 | gbsHWWAMP627; BS00111318_51 | Traes_2AS_F19BE023F | 941,006-945,372 | Disease resistance protein, LRR, NB-ARC, P-loop containing nucleoside triphosphate hydrolase |

LRR, leucine rich repeat; NB-ARC, nucleotide binding-APAF-1 (apoptotic protease-activating factor-1), R proteins and CED-4 (*Caenorhabditis elegans* death-4 protein); PAL, Phenylalanine ammonia-lyase; STPK, serine/threonine-protein kinase.

**Supplementary Table 2: Markers significantly associated with seedling resistance to tan spot**

| Marker | FDR-adjusted p-value | R^2^ | Synonymous/nearest marker | *T. aestivum* gene | Gene location | Domain(s) in *T. aestivum* gene transcript |
| --- | --- | --- | --- | --- | --- | --- |
| S1_3589926 | 8.42E-02 | 0.1 | gbsCNLmaster33207 | Traes_1AS_BF353B963 | 3,421,247-3,425,271 | NBS-LRR type resistance protein |
|  |  |  |  | Traes_1AS_F098402B4 | 3,592,922-3,593,878 | Proteinase inhibitor I12, Bowman-Birk |
| S7_182028651 | 1.36E-01 | 0.08 | WCSS1_contig4364616_3AL-1581 | Traes_3AL_91749D67D | 182,074,241-182,086,701 | Powdery mildew resistance protein, LRR, NB-ARC, P-loop containing nucleoside triphosphate hydrolase |
| S4_239686345 | 1.36E-01 | 0.08 | gbsHWWAMP48121 | Traes_2AL_34A3B95BE | 239,659,644-239,660,447 | Plant disease resistance response protein |
|  |  |  |  |  | 239,659,644-239,660,447 |  |
|  |  |  |  | Traes_2AL_97FC5264A | 239,704,673-239,706,147 | Protein kinase domain, STPK |
| S8_12198705 | 1.36E-01 | 0.08 | gbsHWWAMP48269 | TRAES3BF270500020CFD_g | 12,178,410-12,179,420 |  |
| S8_13415415 | 1.36E-01 | 0.07 | - | TRAES3BF060400190CFD_g | 13,425,488-13,429,397 |  |
| S16_4196814 | 1.36E-01 | 0.07 | gbsHWWAMP26329, IWA3316 | Traes_6AS_3A682BA20 | 4,265,707-4,266,195 | Protein kinase domain, STPK |
| S8_7801088 | 1.36E-01 | 0.07 | gbsHWWAMP35662, CAP8_c1614_529 | TRAES3BF060200040CFD_g | 7,710,250-7,715,050 |  |
|  |  |  |  | TRAES3BF060200010CFD_g | 7,797,083-7,801,241 |  |
| S8_1092429 | 1.76E-01 | 0.06 |  | TRAES3BF035300120CFD_g | 1,087,451-1,090,526 |  |
| S7_4804454 | 1.76E-01 | 0.06 | WCSS1_contig3369578_3AS-225 | Traes_3AS_769E90DDD | 4,631,516-4,633,193 | Protein kinase domain, STPK |
| S16_191519837 | 2.03E-01 | 0.05 | WCSS1_contig5773093_6AL-7687 | Traes_6AL_4815187D4 | 191,517,427-191,519,087 | Concanavalin A-like lectin/glucanase domain, LRR, STPK |
| S1_2331617 | 2.31E-01 | 0.05 | gbsCNLmaster9403 | Traes_1AS_AAB89883E1 | 2,295,827-2,298,821 | LRR, NB-ARC, P-loop containing nucleoside triphosphate hydrolase |
|  |  |  |  | Traes_1AS_459048879 | 2,191,166-2,194,102 |  |
|  |  |  |  | Traes_1AS_3BE2A2127 | 2,416,572-2,418,737 | LRR domain, L domain-like |
| S7_4563676 | 2.31E-01 | 0.05 | gbsCNLmaster41833 | Traes_3AS_817ECEF75 | 4,494,005-4,494,998 | STPK |
| S5_281016023 | 2.52E-01 | 0.05 | WCSS1_contig8006380_2BL-6874 | Traes_2BL_7C2F474DE | 281,027,230-281,028,685 | Plant peroxidase |
|  |  |  |  | Traes_2BL_D055B271C | 281,011,689-281,012,413 | ABC transporter type 1, transmembrane domain |
| S1_2584791 | 2.58E-01 | 0.05 | WCSS1_contig3105145_1AS-1474 | Traes_1AS_C8A8A4118 | 2,583,319-2,585,608 |  |
|  |  |  |  | Traes_1AS_B716E0B0E | 2,564,929-2,568,129 | LRR |
|  |  |  |  | Traes_1AS_8D33AB43B | 2,555,887-2,561,122 | ABC transporter type 1, transmembrane domain, P-loop containing nucleoside triphosphate hydrolase, Peroxisomal fatty acyl CoA transporter |
|  |  |  |  | Traes_1AS_C7A8188D1 | 2,521,366-2,527,020 | Disease resistance protein, Coiled-coils |

ABC, Adenosine triphosphate binding cassette; LRR, leucine rich repeat; NB-ARC, nucleotide binding-APAF-1 (apoptotic protease-activating factor-1), R proteins and CED-4 (*Caenorhabditis elegans* death-4 protein); NBS-LRR, nucleotide binding site-leucine rich repeat; STPK, serine/threonine-protein kinase.

**Supplementary Table 3: Markers significantly associated with seedling and adult plant resistance to stripe rust**

| Dataset | Marker | FDR-adjusted p-value | Marker R^2^ | Synonymous/nearest markers | *T. aestivum* gene | Gene location | Domain(s) in *T. aestivum* gene transcript |
| --- | --- | --- | --- | --- | --- | --- | --- |
| Njoro 2011 | S4_208035 | 1.67E-01 | 0.09 | gbsHWWAMP20269 | Traes_2AS_5EB59FFC0 | 207,328-208,813 | Aromatic amino acid lyase |
| Quito 2012 |  | 1.03E-01 | 0.09 |  |  |  |  |
| Seedling |  | 9.04E-07 | 0.17 |  |  |  |  |
| Toluca 2011 |  | 3.38E-05 | 0.14 |  |  |  |  |
| Toluca 2013 |  | 1.40E-05 | 0.16 |  |  |  |  |
| Njoro 2011 | S4_508877 | 1.67E-01 | 0.09 | gbsHWWAMP30180; BS00022024_51 | Traes_2AS_6A15EE669 | 465,926-467,722 | ABC transporter type 1, transmembrane domain, P-loop containing nucleoside triphosphate hydrolase, AAA+ ATPase domain |
| Quito 2012 |  | 1.27E-04 | 0.14 |  |  |  |  |
| Seedling |  | 6.69E-11 | 0.27 |  |  |  |  |
| Toluca 2011 |  | 1.35E-04 | 0.13 |  |  |  |  |
| Toluca 2013 |  | 2.15E-07 | 0.2 |  |  |  |  |
| Njoro 2011 | S4_944423 | 2.52E-02 | 0.1 | gbsHWWAMP627; BS00111318_51 | Traes_2AS_F19BE023F | 941,006-945,372 | Disease resistance protein, LRR, NB-ARC, P-loop containing nucleoside triphosphate hydrolase |
| Quito 2012 |  | 2.63E-04 | 0.11 |  |  |  |  |
| Seedling |  | 5.18E-10 | 0.24 |  |  |  |  |
| Toluca 2011 |  | 1.21E-04 | 0.13 |  |  |  |  |
| Toluca 2013 |  | 9.69E-08 | 0.2 |  |  |  |  |
| Quito 2012 | S4_5007061 | 3.20E-01 | 0.07 | gbsHWWAMP10929, Tdurum_contig10785_103 | Traes_2AS_6BC67DD45 | 5,005,258-5,009,619 | Leucine-rich repeat domain, L domain-like |
| Seedling |  | 5.48E-05 | 0.17 |  |  |  |  |
| Njoro 2011 | S4_5287800 | 3.28E-02 | 0.1 | gbsHWWAMP47479, BobWhite_c43123_104 | Traes_2AS_A477CDA77 | 5,203,111-5,204,650 | P-loop containing nucleoside triphosphate hydrolase |
| Quito 2012 |  | 1.67E-04 | 0.12 |  |  |  |  |
| Seedling |  | 2.46E-08 | 0.21 |  |  |  |  |
| Toluca 2011 |  | 4.04E-04 | 0.11 |  |  |  |  |
| Toluca 2013 |  | 1.39E-05 | 0.16 |  |  |  |  |
| Njoro 2011 | S4_7117805 | 2.65E-02 | 0.1 | gbsHWWAMP51863,  RAC875_c52554_258 | Traes_2AS_6CE6AB560 | 7,118,296-7,123,325 | Leucine-rich repeat domain, L domain-like, NB-ARC, P-loop containing nucleoside triphosphate hydrolase |
| Quito 2012 |  | 1.26E-03 | 0.1 |  |  |  |  |
| Seedling |  | 1.44E-08 | 0.21 |  |  |  |  |
| Toluca 2011 |  | 2.45E-03 | 0.11 |  |  |  |  |
| Toluca 2013 |  | 4.75E-06 | 0.16 |  |  |  |  |
| Njoro 2011 | S6_132714407 | 2.55E-01 | 0.05 | gbsHWWAMP22013, BobWhite_c17572_339 | Traes_2DL_4B5D621C1 | 132,713,603-132,717,046 | Concanavalin A-like lectin/glucanase domain, STPK |
| Quito 2012 | S8_17773150 | 6.46E-03 | 0.1 | gbsHWWAMP56788 | TRAES3BF050800140CFD_g | 17,778,840-17,783,220 | Concanavalin A-like lectin/glucanase domain, STPK |
| Toluca 2011 | S8_566227604 | 1.75E-01 | 0.06 | gbsHWWAMP45879, Excalibur_c15332_453 | TRAES3BF027700080CFD_g | 566,221,006-566,227,345 | Concanavalin A-like lectin/glucanase domain, LRR, STPK |
| Toluca 2013 | S21_4853558 | 7.81E-02 | 0.07 | gbsHWWAMP7042 | Traes_7DS_600B0996B | 4,850,870-4,853,045 | Mlo-related protein |

ABC, Adenosine triphosphate binding cassette; LRR, leucine rich repeat; NB-ARC, nucleotide binding-APAF-1 (apoptotic protease-activating factor-1), R proteins and CED-4 (*Caenorhabditis elegans* death-4 protein); NBS-LRR, nucleotide binding site-leucine rich repeat; STPK, serine/threonine-protein kinase.

**Supplementary Table 4: Genes in the 2AS distal chromosomal region (0 to 7,123,325 bp)**

| Gene | Location | Orthologue | Predicted function | Species | Identity | Domain(s) in *T. aestivum* gene transcript |
| --- | --- | --- | --- | --- | --- | --- |
| Traes_2AS_15D7300B6 | 11,485-15,872 | TRIUR3_11874 | Protein EIN4 | *Triticum urartu* | 96 | CheY-like superfamily; GAF domain; Histidine kinase-like ATPase, C-terminal domain. |
| Traes_2AS_BB7AAC01E | 56,682-61,859 |  | FMN-linked oxidoreductases superfamily protein |  |  |  |
| Traes_2AS_88D6A69DD | 78,893-79,492 | F775_15220 | Cytochrome P450 76C1 | *Aegilops tauschii* | 78 | Cytochrome P450 |
| Traes_2AS_E1DD8FB4D | 194,022-195,764 | TRIUR3_10292 | Obtusifoliol 14-alpha demethylase | *Triticum urartu* | 86 | Cytochrome P450 |
| Traes_2AS_821E10536 | 204,922-206,136 | F775_21407 | Cytochrome P450 86A1 | *Aegilops tauschii* | 91 | Cytochrome P450 |
| Traes_2AS_5EB59FFC0 | 207,328-208,813 | F775_06675 | Phenylalanine ammonia-lyase | *Aegilops tauschii* | 92 | Aromatic amino acid lyase |
| Traes_2AS_48D41FC99 | 232,316-233,553 | AT4G26490 | Late embryogenesis abundant (LEA) hydroxyproline-rich glycoprotein family | *Arabidopsis thaliana* | 38 | Late embryogenesis abundant protein, LEA-14 |
| Traes_2AS_174193654 | 281,526-284,506 | ONIVA08G18540 | Peptidase S24/S26A/S26B/S26C family protein | *Oryza nivara* | 45 | Peptidase S24/S26, beta-ribbon domain |
| Traes_2AS_ADA59BD4F | 349,942-355,345 |  | hexokinase 3 |  |  |  |
| Traes_2AS_EA89CC3DF | 356,963-372,324 | GSMUA_Achr9G08570_001 | histone-lysine N-methyltransferase ASHH2 | *Musa acuminata* | 30 | AWS domain, SET domain, Zinc finger, CW-type |
| Traes_2AS_FC98C7EB9 | 430,710-432,296 | GSMUA_AchrUn_randomG05100_001 | Cytochrome P450 89A2 | *Musa acuminata* | 33 | Cytochrome P450 |
| Traes_2AS_6A15EE669 | 465,926-467,722 | LOC100842644 | ABC transporter B family member 4-like | *Brachypodium distachyon* | 100 | ABC transporter type 1, transmembrane domain, P-loop containing nucleoside triphosphate hydrolase, AAA+ ATPase domain |
| Traes_2AS_71B82606A | 506,701-509,179 | GSMUA_Achr8G19340_001 | Membrane related protein CP5 | *M. acuminata* | 66 | START domain |
| Traes_2AS_F485758C1 | 510,835-513,787 |  | pyruvate dehydrogenase E1 alpha |  |  |  |
| Traes_2AS_D917848B7 | 680,822-686,162 |  | D111/G-patch domain-containing protein |  |  |  |
| Traes_2AS_33FC65871 | 685,917-691,199 |  | Eukaryotic initiation factor 3 gamma subunit family protein |  |  |  |
| Traes_2AS_D2D7342B3 | 723,415-724,755 | TRIUR3_14654 | Cytochrome P450 86B1 | *Triticum urartu* | 97 | Cytochrome P450 |
| Traes_2AS_21DD59F60 | 787,888-788,679 | MTR_3g009500 | UDP-glucosyltransferase | *Medicago truncatula* | 27 | UDP-glucuronosyl/UDP-glucosyltransferase |
| Traes_2AS_1B39D2FA8 | 812,955-814,381 |  |  |  |  | Domain of unknown function DUF4371, Ribonuclease H-like domain |
| Traes_2AS_09C4D333F | 893,817-895,658 | F775_15920 | Putative NAD(P)H-dependent oxidoreductase 1 | *Aegilops tauschii* | 95 | Aldo/keto reductase, NADP-dependent oxidoreductase domain |
| Traes_2AS_FEFDC29F2 | 900,479-905,272 |  |  |  |  | Histone deacetylase complex subunit SAP30/SAP30-like |
| Traes_2AS_F19BE023F | 941,006-945,372 | TRIUR3_09185 | Putative disease resistance protein RXW24L | *Triticum urartu* | 92 |  |
| Traes_2AS_C075AEE6C | 1,006,381-1,007,592 | TRIUR3_10294 | Cytochrome P450 76C2 | *Triticum urartu* | 82 | Cytochrome P450 |
| Traes_2AS_048E13951 | 1,016,839-1,021,303 |  |  |  |  | Protein phosphatase 2C |
| Traes_2AS_DE0A69F86 | 1,029,392-1,031,392 | F775_15954 | Cytochrome P450 86A1 | *Aegilops tauschii* | 54 | Cytochrome P450 |
| Traes_2AS_9A9E4CC41 | 1,040,393-1,040,677 |  |  |  |  | HAT dimerisation domain, C-terminal, Ribonuclease H-like domain |
| Traes_2AS_ACFF3CB9F | 1,064,508-1,065,138 |  |  |  |  | NB-ARC |
| Traes_2AS_AFEB22E37 | 1,078,143-1,079,932 |  |  |  |  | P-loop containing nucleoside triphosphate hydrolase |
| Traes_2AS_FA7C1F225 | 1,125,533-1,127,020 | TCM_015276 | UDP-glucosyl transferase 88A1, putative | *Theobroma cacao* | 52 | UDP-glucuronosyl/UDP-glucosyltransferase |
| Traes_2AS_A087099AF | 1,183,721-1,186,448 |  |  |  |  |  |
| Traes_2AS_D57565E23 | 1,186,917-1,192,080 |  |  |  |  |  |
| Traes_2AS_9F5A6C54F | 1,199,816-1,201,857 | OS01G0804900 | Cytochrome P450-dependent fatty acid hydroxylase-like protein | *Oryza sativa Japonica* | 64 | Cytochrome P450 |
| Traes_2AS_7AE2FEA16 | 1,211,604-1,212,557 |  |  |  |  | Uncharacterised protein family UPF0546 |
| Traes_2AS_E82763741 | 1,283,975-1,284,799 | GSMUA_Achr2G10160_001 | Putative Cytochrome P450 86B1 | *Musa acuminata* | 32 | Cytochrome P450 |
| Traes_2AS_5BA299224 | 1,306,297-1,309,224 |  |  |  |  | Major facilitator superfamily domain, Proton-dependent oligopeptide transporter family |
| Traes_2AS_6EB8AD265 | 1,345,642-1,348,508 |  |  |  |  | Leucine-rich repeat domain, L domain-like |
| Traes_2AS_6DEE8FF80 | 1,386,842-1,388,352 | TRIUR3_07758 | Cytochrome P450 86B1 | *Triticum urartu* | 84 | Cytochrome P450 |
| Traes_2AS_D2DB0E357 | 1,413,392-1,425,005 | GSMUA_Achr4G29640_001 | Polycomb group protein EMBRYONIC FLOWER 2 | *Musa acuminata* | 32 | Polycomb protein, VEFS-Box |
| Traes_2AS_AC1EE71AB | 1,471,456-1,472,041 |  |  |  |  | Protein of unknown function DUF594 |
| Traes_2AS_7581E280A | 1,506,343-1,511,761 | TRIUR3_01916 | ABC transporter B family member 11 | *Triticum urartu* | 90 | ABC transporter type 1, transmembrane domain, P-loop containing nucleoside triphosphate hydrolase, AAA+ ATPase domain |
| Traes_2AS_00F345CBB | 1,515,753-1,517,096 |  |  |  |  |  |
| Traes_2AS_6F531FBB5 | 1,524,389-1,528,207 f |  |  |  |  | ARID DNA-binding domain, ELM2 domain |
| Traes_2AS_5CAF7A3671 | 1,608,937-1,610,457 | TRIUR3_10228 | Putative disease resistance protein RGA3 | *Triticum urartu* | 99 | NB-ARC, P-loop containing nucleoside triphosphate hydrolase |
| Traes_2AS_0898755211 | 1,613,015-1,614,568 | TRIUR3_10229 | Putative disease resistance RPP13-like protein 1 | *Triticum urartu* | 100 | P-loop containing nucleoside triphosphate hydrolase |
| Traes_2AS_6E1B0E6EF | 1,619,489-1,624,160 | TRIUR3_11002 | Disease resistance protein RGA2 | *Triticum urartu* | 86 | P-loop containing nucleoside triphosphate hydrolase |
| Traes_2AS_5A921E630 | 1,642,311-1,645,309 | TRIUR3_00568 | Sec23/Sec24 protein transport family protein | *Triticum urartu* | 90 | ADF-H/Gelsolin-like domain, Gelsolin-like domain, Sec23/Sec24 beta-sandwich |
| Traes_2AS_6BB5BCCA2 | 1,668,886-1,673,001 | TRIUR3_00558 | Werner Syndrome-like exonuclease | *Triticum urartu* | 88 | Ribonuclease H-like domain |
| Traes_2AS_E1C66CA12 | 1,673,131-1,676,858 |  |  |  |  | Cytochrome oxidase assembly protein 1 |
| Traes_2AS_5BE64EB9E | 1,768,877-1,769,792 |  |  |  |  | Cytochrome P450 |
| Traes_2AS_D66FB2E90 | 1,775,306-1,775,420 |  |  |  |  | Transcription factor TGA like domain |
| Traes_2AS_5A9348E72 | 1,836,189-1,840,014 |  |  |  |  |  |
| Traes_2AS_B5D438C6C | 1,852,098-1,855,412 |  |  |  |  | Protein of unknown function DUF724, Tudor-like, plant |
| Traes_2AS_A8793AD4F | 1,873,413-1,878,909 | TCM_006167 | Yth domain-containing protein, putative isoform 1 | *Theobroma cacao* | 26 | YTH domain |
| Traes_2AS_E82A7163B | 1,880,594-1,884,410 |  |  |  |  | Protein of unknown function DUF1644, Zinc finger, RING/FYVE/PHD-type |
| Traes_2AS_1E8BDB2C6 | 1,896,429-1,902,311 | TRIUR3_01918 | ABC transporter B family member 4 | *Triticum urartu* | 99 | ABC transporter type 1, transmembrane domain, P-loop containing nucleoside triphosphate hydrolase, AAA+ ATPase domain |
| Traes_2AS_B264257CD | 1,916,608-1,917,879 | TRIUR3_00556 | Flavin-containing monooxygenase YUCCA8 | *Triticum urartu* | 100 | Pyridine nucleotide-disulphide oxidoreductase, FAD/NAD(P)-binding domain |
| Traes_2AS_D0C21ADB5 | 1,937,418-1,938,543 |  |  |  |  | DNA-binding WRKY |
| Traes_2AS_5CAF7A367 | 1,977,217-1,978,737 | TRIUR3_10228 | Putative disease resistance protein RGA3 | *Triticum urartu* | 99 | NB-ARC, P-loop containing nucleoside triphosphate hydrolase |
| Traes_2AS_089875521 | 1,981,295-1,982,848 | TRIUR3_10229 | Putative disease resistance RPP13-like protein 1 | *Triticum urartu* | 100 | P-loop containing nucleoside triphosphate hydrolase |
| Traes_2AS_EFB7DF837 | 1,987,769-1,992,440 | TRIUR3_11002 | Disease resistance protein RGA2 | *Triticum urartu* | 87 | P-loop containing nucleoside triphosphate hydrolase |
| Traes_2AS_5C5EAEAB8 | 2,051,212-2,053,364 | TRIUR3_01915 | Tyrosine N-monooxygenase | *Triticum urartu* | 98 | Cytochrome P450 |
| Traes_2AS_B096B5D19 | 2,059,096-2,059,334 |  |  |  |  |  |
| Traes_2AS_9ED98D871 | 2,114,563-2,115,975 | BRADI5G01280 | Sec23/Sec24 protein transport family protein | *Brachypodium distachyon* | 64 | Sec23/Sec24, trunk domain, von Willebrand factor, type A |
| Traes_2AS_7B74BE6D6 | 2,179,779-2,183,218 | F775_25605 | Putative LRR receptor-like serine/threonine-protein kinase | *Aegilops tauschii* | 96 | Concanavalin A-like lectin/glucanase domain, Leucine-rich repeat, Serine/threonine-protein kinase |
| Traes_2AS_0C6E89B86 | 2,185,152-2,193,592 |  | UDP-Glycosyltransferase superfamily protein |  |  | UDP-Glycosyltransferase |
| Traes_2AS_6E3F18D20 | 2,328,227-2,333,933 | BRADI5G01220 | AAR2 protein family | *Brachypodium distachyon* | 90 | A1 cistron-splicing factor, AAR2 |
| Traes_2AS_1171AE3E3 | 2,368,039-2,368,281 |  |  |  |  |  |
| Traes_2AS_90E3B2E76 | 2,371,741-2,378,409 | TRIUR3_00568 | Sec23/Sec24 protein transport family protein | *Triticum urartu* | 98 | ADF-H/Gelsolin-like domain, Gelsolin-like domain, Sec23/Sec24 beta-sandwich, Sec23/Sec24, helical domain, Sec23/Sec24, trunk domain, Zinc finger, Sec23/Sec24-type, von Willebrand factor, type A |
| Traes_2AS_D38E1E530 | 2,425,561-2,429,488 | TRIUR3_33851 | Cytochrome P450 85A1 | *Triticum urartu* | 97 | Cytochrome P450 |
| Traes_2AS_1493D0ADB | 2,434,789-2,438,797 | TRIUR3_16698 | TOM1-like protein 2 | *Triticum urartu* | 100 | ENTH/VHS, GAT domain, VHS domain, Target of Myb protein 1 |
| Traes_2AS_D2FB48992 | 2,511,299-2,515,640 | TRIUR3_01922 | Arginine/serine-rich-splicing factor RSP31 | *Triticum urartu* | 97 | Nucleotide-binding alpha-beta plait domain, RNA recognition motif domain |
| Traes_2AS_D3715AC4A | 2,600,647-2,602,717 | BRADI2G40400 | alpha/beta-Hydrolases superfamily protein | *Brachypodium distachyon* | 71 | Alpha/Beta hydrolase fold, Dienelactone hydrolase |
| Traes_2AS_48DA475B0 | 2,605,114-2,608,922 | POPTR_0008s20920 | NAD(P)-binding Rossmann-fold superfamily protein | *Populus trichocarpa* | 68 | NAD(P)-binding domain |
| Traes_2AS_1FB8F6760 | 2,610,953-2,611,098 | F775_30926 | Nicotianamine synthase-like 5 protein | *Aegilops tauschii* | 100 | Nicotianamine synthase |
| Traes_2AS_AFA56788E | 2,631,651-2,635,406 | OS03G0602300 | Cytochrome P450 85A1 | *Oryza sativa Japonica* | 84 | Cytochrome P450 |
| Traes_2AS_C8325AEE0 | 2,672,752-2,673,312 |  |  |  |  |  |
| Traes_2AS_0E7069178 | 2,682,863-2,688,664 | TRIUR3_15844 | E3 ubiquitin ligase BIG BROTHER-related protein | *Triticum urartu* | 99 | Zinc finger, RING-type |
| Traes_2AS_F8779F643 | 2,708,791-2,716,281 |  |  |  |  | Serine/threonine-protein kinase |
| Traes_2AS_27D2CB02A | 2,735,584-2,743,313 | TRIUR3_03051 | Mortality factor 4-like protein 1 | *Triticum urartu* | 71 | Chromo domain-like, MRG domain |
| Traes_2AS_18956EE53 | 2,744,835-2,746,252 | GSMUA_Achr4G26690_001 | pectate lyase 15 | *Musa acuminata* | 85 | Pectate lyase, AmbAllergen |
| Traes_2AS_0C6583718 | 2,746,355-2,748,906 | GRMZM2G157354 | Nicotiana lesion-inducing like protein | *Zea mays* | 57 | HR-like lesion-inducer |
| Traes_2AS_D35789679 | 2,781,055-2,784,866 | TCM_001771 | 4-hydroxyphenylacetaldehyde oxime monooxygenase, putative | *Theobroma cacao* | 51 | Cytochrome P450 |
| Traes_2AS_7E07335F8 | 2,809,078-2,810,795 | F775_17719 | Tyrosine N-monooxygenase | *Aegilops tauschii* | 94 | Cytochrome P450 |
| Traes_2AS_1E76C16FF | 2,824,218-2,826,100 |  |  |  |  | P-loop containing nucleoside triphosphate hydrolase |
| Traes_2AS_ECCD1E063 | 2,827,522-2,831,276 | GSMUA_Achr3G09670_001 | ABC transporter B family member | *Musa acuminata* | 65 | ABC transporter type 1, transmembrane domain |
| Traes_2AS_D7F13D5C9 | 2,831,827-2,833,805 | POPTR_0001s29260 | fatty acid amide hydrolase | *Populus trichocarpa* | 49 | Amidase signature domain |
| Traes_2AS_BBE5262D9 | 2,838,542-2,840,321 |  |  |  |  |  |
| Traes_2AS_A8BF6F543 | 2,845,738-2,847,421 |  | heat stable protein 1 |  |  | Dimeric alpha-beta barrel, Stress responsive alpha-beta barrel |
| Traes_2AS_C8DB2D8D6 | 2,857,320-2,859,807 | F775_01098 | Cysteine-rich receptor-like protein kinase 10 | *Aegilops tauschii* | 82 | Serine/threonine-protein kinase |
| Traes_2AS_802012621 | 2,876,318-2,878,062 | TRIUR3_22387 | Wall-associated receptor kinase 3 | *Triticum urartu* | 95 | Concanavalin A-like lectin/glucanase domain, EGF-like calcium-binding domain, Insulin-like growth factor binding protein, N-terminal, Serine/threonine-protein kinase |
| Traes_2AS_5907AC060 | 2,902,811-2,903,319 |  |  |  |  |  |
| Traes_2AS_55A5C5791 | 2,920,103-2,920,895 | Bo9g059980 | FAD-binding Berberine family protein | *Brassica oleraceae* | 43 | Berberine/berberine-like |
| Traes_2AS_55FB06D16 | 2,929,728-2,930,679 | GRMZM2G124785 | Nicotianamine synthase 2 | *Zea mays* | 41 | Nicotianamine synthase |
| Traes_2AS_A3F743F75 | 2,942,903-2,943,796 |  |  |  |  |  |
| Traes_2AS_2E21BFFFB | 2,951,179-2,953,372 | F775_17990 | Chalcone synthase | *Aegilops tauschii* | 94 | Chalcone/stilbene synthase, Polyketide synthase, type III, Thiolase like |
| Traes_2AS_57DFD533D | 2,983,516-2,984,167 |  |  |  |  |  |
| Traes_2AS_8770C8C36 | 3,003,227-3,003,474 |  |  |  |  |  |
| Traes_2AS_6C89A3064 | 3,014,100-3,020,389 |  | ELMO/CED-12 family protein |  |  | Engulfment/cell motility, ELMO |
| Traes_2AS_08A91D578 | 3,021,419-3,021,957 |  |  |  |  |  |
| Traes_2AS_B96BB2490 | 3,031,360-3,038,368 |  | zinc finger (CCCH-type) family protein |  |  | (Uracil-5)-methyltransferase family, Nucleotide-binding alpha-beta plait domain, RNA methyltransferase TrmA, active site, S-adenosyl-L-methionine-dependent methyltransferase |
| Traes_2AS_F2A8F88A3 | 3,062,168-3,063,407 | OS11G0432900 | Serine carboxypeptidase family protein | *Oryza sativa Japonica* | 76 | Alpha/Beta hydrolase fold, Peptidase S10, serine carboxypeptidase |
| Traes_2AS_512E437B5 | 3,070,839-3,071,330 | F775_14818 | Cytochrome P450 71C2 | *Aegilops tauschii* | 98 | Cytochrome P450 |
| Traes_2AS_A3B0031A6 | 3,072,133-3,073,639 | F775_14818 | Cytochrome P450 71C2 | *Aegilops tauschii* | 96 | Cytochrome P450 |
| Traes_2AS_AC62A3F1C | 3,080,626-3,083,718 |  |  |  |  | Domain of unknown function DUF4220 |
| Traes_2AS_7D25F69CB | 3,132,390-3,135,807 | PRUPE_ppa008443mg | Nucleotide-diphospho-sugar transferase family protein | *Prunus persica* | 22 | Nucleotide-diphospho-sugar transferase |
| Traes_2AS_FD9DC88CA1 | 3,147,755-3,150,987 | BRADI5G02990 | FKBP-like peptidyl-prolyl cis-trans isomerase family protein | *Brachypodium distachyon* | 73 | Peptidyl-prolyl cis-trans isomerase, FKBP-type |
| Traes_2AS_0B776BDEE | 3,161,720-3,162,254 |  |  |  |  | Cytochrome P450 |
| Traes_2AS_592926586 | 3,247,963-3,249,013 |  |  |  |  | D-isomer specific 2-hydroxyacid dehydrogenase, NAD-binding domain, NAD(P)-binding domain |
| Traes_2AS_B8091AC54 | 3,300,384-3,305,959 | TRIUR3_23980 | Disease resistance protein RPM1 | *Triticum urartu* | 71 | Leucine-rich repeat domain, L domain-like, NB-ARC, P-loop containing nucleoside triphosphate hydrolase |
| Traes_2AS_5A0BCB6E0 | 3,306,182-3,311,622 |  |  |  |  |  |
| Traes_2AS_32405E54F | 3,314,219-3,318,690 |  | Formamidopyrimidine-DNA glycosylase | *Aegilops tauschii* | 85 | DNA glycosylase/AP lyase, H2TH DNA-binding, Ribosomal protein S13-like, H2TH |
| Traes_2AS_A4BAAEE05 | 3,350,821-3,353,514 | OS04G0178300 | Syn-copalyl diphosphate synthase | *Oryza sativa Japonica* | 65 | Terpene synthase, Terpenoid cyclases/protein prenyltransferase alpha-alpha toroid |
| Traes_2AS_E6FF6A652 | 3,360,901-3,361,678 | BRADI4G17230 | Chalcone and stilbene synthase family protein | *Brachypodium distachyon* | 89 | Chalcone/stilbene synthase, C-terminal, Thiolase like |
| Traes_2AS_972F2CECA | 3,399,290-3,408,485 | TRIUR3_02866 | Cullin-associated NEDD8-dissociated protein 1 | *Triticum urartu* | 94 | Armadillo-like helical, TATA-binding protein interacting (TIP20) |
| Traes_2AS_86E424632 | 3,417,793-3,420,233 | TRIUR3_19384 | Myrcene synthase, chloroplastic | *Triticum urartu* | 86 | Isoprenoid synthase domain, Terpene synthase, N-terminal domain, Terpenoid cyclases/protein prenyltransferase alpha-alpha toroid |
| Traes_2AS_A0DB10548 | 3,420,922-3,423,704 | GSMUA_Achr10G09080_001 | CDT1A - Putative DNA replication initiation protein, expressed | *Musa acuminata* | 46 | CDT1 Geminin-binding domain-like, DNA replication factor Cdt1, C-terminal, Winged helix-turn-helix DNA-binding domain |
| Traes_2AS_AD32B0419 | 3,425,483-3,427,078 | TRIUR3_28181 | Reticuline oxidase-like protein | *Triticum urartu* | 50 | Berberine/berberine-like, CO dehydrogenase flavoprotein-like, FAD-binding, subdomain 2, FAD linked oxidase, N-terminal, FAD-binding, type 2 |
| Traes_2AS_607E72B1A | 3,482,676-3,484,835 | F775_13064 | 1-aminocyclopropane-1-carboxylate oxidase-1-like protein | *Aegilops tauschii* | 96 | Isopenicillin N synthase-like, Non-haem dioxygenase N-terminal domain, Oxoglutarate/iron-dependent dioxygenase |
| Traes_2AS_0FA7292A9 | 3,513,535-3,528,917 |  |  |  |  |  |
| Traes_2AS_D38A5E045 | 3,531,826-3,536,363 | F775_05049 | Putative amidase | *Aegilops tauschii* | 67 | Amidase signature domain |
| Traes_2AS_E5D366E76 | 3,542,721-3,544,462 |  |  |  |  | Cytochrome P450 |
| Traes_2AS_03158984F | 3,621,705-3,622,356 |  |  |  |  |  |
| Traes_2AS_737B8B16E | 3,680,868-3,681,101 |  |  |  |  |  |
| Traes_2AS_195F8322C | 3,696,207-3,697,724 |  |  |  |  |  |
| Traes_2AS_8588747E6 | 3,721,533-3,724,798 |  |  |  |  |  |
| Traes_2AS_292D0991C | 3,764,073-3,765,666 |  |  |  |  | Major facilitator superfamily domain |
| Traes_2AS_0CABCA673 | 3,780,965-3,785,081 | F775_20787 | ABC transporter C family member 4 | *Aegilops tauschii* | 82 | ABC transporter type 1, transmembrane domain, P-loop containing nucleoside triphosphate hydrolase, AAA+ ATPase domain |
| Traes_2AS_F15D136CF | 3,811,693-3,812,895 | TRIUR3_03937 | Putative glutathione S-transferase GSTF1 | *Triticum urartu* | 95 | Glutathione S-transferase, Thioredoxin-like fold |
| Traes_2AS_712C217B9 | 3,822,679-3,823,672 |  |  |  |  |  |
| Traes_2AS_EDB52A1EB | 3,825,633-3,827,560 | F775_01485 | Protein RUPTURED POLLEN GRAIN 1 | *Aegilops tauschii* | 70 | SWEET sugar transporter |
| Traes_2AS_E3638EE97 | 3,962,722-3,963,023 |  |  |  |  |  |
| Traes_2AS_9D1FC09D7 | 3,978,834-3,982,971 |  |  |  |  | Leucine-rich repeat domain, L domain-like, NB-ARC, P-loop containing nucleoside triphosphate hydrolase |
| Traes_2AS_0C03C4F29 | 3,993,406-3,993,989 | TRIUR3_06927 | Defensin-like protein 2 | *Triticum urartu* | 93 | Gamma Purothionin, Knottin, scorpion toxin-like |
| Traes_2AS_37A335CE4 | 3,999,946-4,002,582 | F775_02916 | Formin-like protein 11 | *Aegilops tauschii* | 94 | Formin, FH2 domain |
| Traes_2AS_29CF270C8 | 4,055,542-4,056,267 | F775_14593 | Defensin-like protein P322 | *Aegilops tauschii* | 95 | Gamma Purothionin, Knottin, scorpion toxin-like |
| Traes_2AS_0E33CD71F | 4,131,971-4,132,441 |  |  |  |  | Nucleotide-diphospho-sugar transferase |
| Traes_2AS_0E225338F | 4,185,852-4,186,590 | F775_11453 | Chemocyanin | *Aegilops tauschii* | 72 | Cupredoxin, Plastocyanin-like |
| Traes_2AS_484015532 | 4,217,044-4,217,949 |  |  |  |  |  |
| Traes_2AS_49F9C2E5D | 4,249,429-4,252,279 |  |  |  |  | Peptidase C65, otubain |
| Traes_2AS_453C10ECC | 4,273,730-4,291,014 | Si009627m.g | fatty acid amide hydrolase | *Setaria italica* | 82 | Amidase |
| Traes_2AS_3F13B523F | 4,324,394-4,325,943 | Bo8g116100 | splicing factor Prp18 family protein | *Brassica oleraceae* | 78 | Prp18 |
| Traes_2AS_82FF45D92 | 4,360,958-4,361,268 |  |  |  |  | CO dehydrogenase flavoprotein-like, FAD-binding, subdomain 2, FAD linked oxidase, N-terminal, FAD-binding, type 2 |
| Traes_2AS_C913A8043 | 4,470,322-4,475,786 | TRIUR3_14868 | G-type lectin S-receptor-like serine/threonine-protein kinase | *Triticum urartu* | 99 | Bulb-type lectin domain, Concanavalin A-like lectin/glucanase domain, Serine/threonine-protein kinase, |
| Traes_2AS_1D2CDFD90 | 4,490,058-4,490,782 |  |  |  |  |  |
| Traes_2AS_D61611BDC | 4,539,695-4,540,797 |  |  |  |  |  |
| Traes_2AS_D537D01E3 | 4,602,759-4,603,439 |  |  |  |  |  |
| Traes_2AS_AA9C737C7 | 4,632,431-4,634,488 | F775_13064 | 1-aminocyclopropane-1-carboxylate oxidase-1-like protein | *Aegilops tauschii* | 93 | Isopenicillin N synthase-like, Oxoglutarate/iron-dependent dioxygenase |
| Traes_2AS_786CFD895 | 4,645,645-4,646,384 |  |  |  |  | Glycoside hydrolase |
| Traes_2AS_1A307CE26 | 4,656,211-4,656,893 | OS08G0432300 | Putative teosinte branched1 protein | *Oryza sativa Japonica* | 53 | Transcription factor TCP subgroup |
| Traes_2AS_768355513 | 4,674,801-4,676,429 | TRIUR3_13099 | 1-aminocyclopropane-1-carboxylate oxidase-like protein 2 | *Triticum urartu* | 62 | Isopenicillin N synthase-like, Non-haem dioxygenase N-terminal domain, Oxoglutarate/iron-dependent dioxygenase |
| Traes_2AS_4FEC7A465 | 4,699,741-4,704,668 | F775_17871 | Syn-copalyl diphosphate synthase | *Aegilops tauschii* | 74 | Isoprenoid synthase domain, Terpene synthase, Terpenoid cyclases/protein prenyltransferase alpha-alpha toroid |
| Traes_2AS_04D7D387C | 4,776,276-4,777,628 |  |  |  |  |  |
| Traes_2AS_CAA2FBF93 | 4,882,731-4,886,348 | TRIUR3_08998 | Beta-glucosidase 16 | *Triticum urartu* | 88 | Glycoside hydrolase |
| Traes_2AS_67817FEBC | 4,891,171-4,894,255 | GSMUA_Achr1G09280_001 | serine-type peptidase | *Musa acuminata* | 63 | Peptidase |
| Traes_2AS_6D59C67F0 | 4,900,479-4,915,398 | GRMZM2G154509 | Phosphoglycerate mutase-like protein isoform 1 | *Zea mays* | 72 | Histidine phosphatase superfamily |
| Traes_2AS_AD1248C31 | 4,921,435-4,921,732 |  |  |  |  | Chloramphenicol acetyltransferase-like domain |
| Traes_2AS_2EB281841 | 4,937,584-4,941,371 | OS10G0124500 | F-box domain containing protein | *Oryza sativa Japonica* | 51 | F-box domain |
| Traes_2AS_09E707C65 | 4,948,052-4,951,281 | TRIUR3_12520 | Cysteine-rich receptor-like protein kinase 41 | *Triticum urartu* | 82 | Concanavalin A-like lectin/glucanase domain, MSP domain, PapD-like, Protein kinase |
| Traes_2AS_E0AFA6D18 | 4,972,252-4,975,044 | F775_14936 | Bifunctional 3'-phosphoadenosine 5'-phosphosulfate synthetase 2 | *Aegilops tauschii* | 96 | ATP-sulfurylase PUA-like domain, PUA-like domain, Rossmann-like alpha/beta/alpha sandwich fold, Sulphate adenylyltransferase |
| Traes_2AS_6BC67DD45 | 5,005,258-5,009,619 | TRIUR3_16539 | Putative disease resistance RPP13-like protein 1 | *Triticum urartu* | 97 | Leucine-rich repeat domain, L domain-like |
| Traes_2AS_FBBAC0883 | 5,092,563-5,096,155 | GSMUA_Achr2G11220_001 | Putative amidase | *Musa acuminata* | 61 | Amidase |
| Traes_2AS_82A750758 | 5,104,285-5,107,870 | TRIUR3_01665 | Putative acetyl-CoA acetyltransferase, cytosolic 2 | *Triticum urartu* | 91 | Thiolase-like |
| Traes_2AS_A9F768C2B | 5,111,270-5,112,120 | GRMZM2G068947 | 12-oxo-phytodienoic acid reductase | *Zea mays* | 44 | Aldolase-type TIM barrel, NADH:flavin oxidoreductase/NADH oxidase, N-terminal |
| Traes_2AS_52D58DF7F | 5,112,603-5,114,095 | TCM_046775 | 2-oxoglutarate (2OG) and Fe(II)-dependent oxygenase superfamily protein | *Theobroma cacao* | 57 | Isopenicillin N synthase-like, Oxoglutarate/iron-dependent dioxygenase |
| Traes_2AS_74C2A5D1F | 5,139,357-5,141,697 | TRIUR3_20816 | Disease resistance RPP13-like protein 4 | *Triticum urartu* | 40 | Leucine-rich repeat domain, L domain-like |
| Traes_2AS_A477CDA77 | 5,203,111-5,204,650 | TRIUR3_30356 | Putative disease resistance RPP13-like protein 1 | *Triticum urartu* | 71 | P-loop containing nucleoside triphosphate hydrolase |
| Traes_2AS_C475CC0F9 | 5,284,999-5,288,091 | F775_11445 | Vacuolar amino acid transporter 1 | *Aegilops tauschii* | 98 | Amino acid transporter, transmembrane |
| Traes_2AS_8228ECD84 | 5,326,512-5,327,522 | MLOC_54115 | peptidylprolyl cis/trans isomerase, NIMA-interacting 1 | *Hordeum vulgare* | 69 | Peptidyl-prolyl cis-trans isomerase, PpiC-type |
| Traes_2AS_FD956B4EF | 5,347,125-5,348,654 | MTR_4g128210 | muniscin carboxy-terminal mu-like domain protein | *Medicago truncatula* | 55 | Mu homology domain, Muniscin C-terminal |
| Traes_2AS_7A4976FA9 | 5,360,566-5,363,634 | GSMUA_Achr11G02290_001 | CBS domain containing membrane protein | *Musa acuminata* | 51 | CBS domain |
| Traes_2AS_FEBDEF579 | 5,397,058-5,397,826 |  |  |  |  |  |
| Traes_2AS_F06C61AB1 | 5,429,704-5,431,056 |  |  |  |  | P-loop containing nucleoside triphosphate hydrolase |
| Traes_2AS_38949B685 | 5,437,919-5,441,973 | F775_06790 | Serine carboxypeptidase-like 19 | *Aegilops tauschii* | 90 | Alpha/Beta hydrolase fold, Peptidase S10, serine carboxypeptidase |
| Traes_2AS_03F50010A | 5,469,915-5,470,507 |  |  |  |  |  |
| Traes_2AS_85E4E40C3 | 5,530,550-5,532,694 | F775_00200 | Cytokinin-O-glucosyltransferase 2 | *Aegilops tauschii* | 55 | UDP-glucuronosyl/UDP-glucosyltransferase |
| Traes_2AS_54398D868 | 5,551,825-5,553,397 |  |  |  |  | P-loop containing nucleoside triphosphate hydrolase |
| Traes_2AS_28B3FAEB3 | 5,557,208-5,561,275 | OS12G0123500 | Probable apyrase 3 | *Oryza sativa Japonica* | 70 | Nucleoside phosphatase GDA1/CD39 |
| Traes_2AS_2821A7128 | 5,584,894-5,586,491 | F775_15232 | Anthranilate N-benzoyltransferase protein 1 | *Aegilops tauschii* | 72 | Chloramphenicol acetyltransferase-like domain, transferse |
| Traes_2AS_F25B2DA46 | 5,620,118-5,621,330 |  |  |  |  |  |
| Traes_2AS_CED8C9B73 | 5,642,653-5,650,977 | F775_01103 | Putative histone acetyltransferase HAC-like protein 3 | *Aegilops tauschii* | 76 | CBP/p300-type histone acetyltransferase domain, Histone H3-K56 acetyltransferase, RTT109, Zinc finger, FYVE/PHD-type, Zinc finger, TAZ-type, Zinc finger, ZZ-type |
| Traes_2AS_3D0A18D67 | 5,660,860-5,663,270 |  |  |  |  | Glycosyl transferase, family 43, Nucleotide-diphospho-sugar transferases |
| Traes_2AS_D11AD107B | 5,664,472-5,667,082 | GSMUA_Achr9G10980_001 | Putative Eukaryotic translation initiation factor 3 subunit M | *Musa acuminata* | 64 | Proteasome component (PCI) domain, Winged helix-turn-helix DNA-binding domain |
| Traes_2AS_BA5EB39CB | 5,794,485-5,796,380 | OS08G0157500 | Flavone 3'-O-methyltransferase 1 | *Oryza sativa Japonica* | 65 | O-methyltransferase COMT-type, Plant methyltransferase dimerisation, S-adenosyl-L-methionine-dependent methyltransferase, Winged helix-turn-helix DNA-binding domain |
| Traes_2AS_B78968A63 | 5,812,028-5,821,870 | F775_28219 | Cyclopropane-fatty-acyl-phospholipid synthase | *Aegilops tauschii* | 91 | Amine oxidase, Pyridine nucleotide-disulphide oxidoreductase, FAD/NAD(P)-binding domain 3.50.50.60, S-adenosyl-L-methionine-dependent methyltransferase |
| Traes_2AS_8D7326365 | 5,878,646-5,880,443 | F775_12427 | Protein RUPTURED POLLEN GRAIN 1 | *Aegilops tauschii* | 98 | SWEET sugar transporter |
| Traes_2AS_43F1E3236 | 5,889,641-5,890,252 |  |  |  |  |  |
| Traes_2AS_C6EE01E7C | 5,925,019-5,925,190 |  |  |  |  | DNA glycosylase/AP lyase, catalytic domain |
| Traes_2AS_11CD58DCB | 5,960,922-5,961,200 |  |  |  |  |  |
| Traes_2AS_7D58356F2 | 5,984,614-5,988,797 | LOC100830175 | Disease resistance protein RGA3 | *Brachypodium distachyon* | 79 | Leucine-rich repeat domain, L domain-like, NB-ARC, P-loop containing nucleoside triphosphate hydrolase |
| Traes_2AS_0F0720621 | 6,022,294-6,025,479 | F775_04990 | Wall-associated receptor kinase 2 | *Aegilops tauschii* | 92 | EGF-like calcium-binding domain, EGF-like calcium-binding, conserved site, Insulin-like growth factor binding protein, N-terminal, Serine-threonine/tyrosine-protein kinase catalytic domain |
| Traes_2AS_892049962 | 6,028,623-6,030,299 | GSMUA_Achr9G17450_001 | Sugar transporter ERD6-like 5 | *Musa acuminata* | 71 | General substrate transporter, Major facilitator superfamily domain |
| Traes_2AS_F3D9CA560 | 6,041,291-6,046,379 | BRADI5G01960 | zinc finger (Ran-binding) family protein | *Brachypodium distachyon* | 79 | Zinc finger, RanBP2-type |
| Traes_2AS_E55705603 | 6,047,423-6,049,703 | GSMUA_Achr7G16470_001 | Probable carbohydrate esterase At4g34215 | *Musa acuminata* | 48 | SGNH hydrolase-type esterase domain |
| Traes_2AS_9C459F3AA | 6,051,185-6,056,833 | F775_26585 | Pumilio-like protein | *Aegilops tauschii* | 96 | Armadillo-like helical, Pumilio RNA-binding repeat |
| Traes_2AS_84673C57E | 6,115,027-6,116,935 | GSMUA_Achr3G25740_001 | Serine carboxypeptidase-like 18 | *Musa acuminata* | 45 | Alpha/Beta hydrolase fold, Peptidase S10, serine carboxypeptidase |
| Traes_2AS_1742B494A | 6,146,931-6,152,135 | TRIUR3_09802 | actin-related protein 9 | *Triticum urartu* | 75 | Actin-related protein 8/Plant actin-related protein 9 |
| Traes_2AS_E899EC1A7 | 6,187,228-6,189,784 |  |  |  |  | Peptidase S54, rhomboid domain, Protein of unknown function DUF1751, integral membrane, eukaryotic |
| Traes_2AS_FD9DC88CA | 6,200,795-6,204,027 | BRADI5G02990 | FKBP-like peptidyl-prolyl cis-trans isomerase family protein | *Brachypodium distachyon* | 73 | Peptidyl-prolyl cis-trans isomerase, FKBP-type |
| Traes_2AS_B0F02BF94 | 6,214,894-6,215,271 | MTR_3g057990 | cytochrome P450 family 71 protein | *Medicago truncatula* | 29 | Cytochrome P450 |
| Traes_2AS_D0CC98BD2 | 6,233,916-6,234,981 | GSMUA_Achr2G11220_001 | Putative amidase C869.01 | *Musa acuminata* | 58 | Amidase |
| Traes_2AS_B1E630276 | 6,264,733-6,268,265 | GRMZM2G164036 | Cytochrome P450 CYP71C36 | *Zea mays* | 57 | Cytochrome P450 |
| Traes_2AS_EF55AF9DD | 6,306,786-6,312,638 | GRMZM2G079538 | Dihydrolipoyllysine-residue succinyltransferase component of 2-oxoglutarate dehydrogenase complex | *Zea mays* | 88 | 2-oxo acid dehydrogenase, lipoyl-binding site, Biotin/lipoyl attachment, Chloramphenicol acetyltransferase-like domain, Dihydrolipoamide succinyltransferase, Single hybrid motif |
| Traes_2AS_3478D9D66 | 6,318,771-6,323,180 | TRIUR3_34055 | Putative disease resistance RPP13-like protein 1 | *Triticum urartu* | 95 | Leucine-rich repeat domain, L domain-like, NB-ARC, P-loop containing nucleoside triphosphate hydrolase |
| Traes_2AS_DF1AB5AC4 | 6,441,240-6,442,023 | AT2G30830 | 2-oxoglutarate (2OG) and Fe(II)-dependent oxygenase superfamily protein | *Arabidopsis thaliana* | 43 | Isopenicillin N synthase-like, Non-haem dioxygenase N-terminal domain |
| Traes_2AS_3947F19A9 | 6,471,670-6,474,448 | TRIUR3_11745 | Disease resistance protein RPP13 | *Triticum urartu* | 81 | Leucine-rich repeat domain, L domain-like, NB-ARC, P-loop containing nucleoside triphosphate hydrolase |
| Traes_2AS_7934B4038 | 6,491,252-6,491,568 | F775_14065 | Putative disease resistance protein | *Aegilops tauschii* | 59 | NB-ARC, P-loop containing nucleoside triphosphate hydrolase |
| Traes_2AS_5815C0679 | 6,547,393-6,547,841 | F775_15110 | Putative amidase | *Aegilops tauschii* | 81 | Amidase |
| Traes_2AS_0EF4A20E2 | 6,548,163-6,548,604 |  |  |  |  | D-isomer specific 2-hydroxyacid dehydrogenase, catalytic domain, NAD(P)-binding domain |
| Traes_2AS_9DF4284D3 | 6,579,460-6,579,783 |  |  |  |  |  |
| Traes_2AS_707CCA800 | 6,587,459-6,589,138 |  |  |  |  | P-loop containing nucleoside triphosphate hydrolase |
| Traes_2AS_537A8D6B5 | 6,646,448-6,652,586 | AT5G60740 | ABC transporter G family member 28 | *Arabidopsis thaliana* | 53 | AAA+ ATPase domain, ABC transporter-like, P-loop containing nucleoside triphosphate hydrolase |
| Traes_2AS_148383FB6 | 6,712,387-6,715,389 | F775_03222 | Putative amidase | *Aegilops tauschii* | 93 | Amidase |
| Traes_2AS_B6A9A23D7 | 6,755,925-6,757,154 | TRIUR3_10639 | 1-aminocyclopropane-1-carboxylate oxidase-like protein 11 | *Triticum urartu* | 93 | Isopenicillin N synthase-like, Oxoglutarate/iron-dependent dioxygenase |
| Traes_2AS_3E73A7BD8 | 6,799,897-6,802,578 | F775_12436 | Protein RUPTURED POLLEN GRAIN 1 | *Aegilops tauschii* | 88 | *SWEET sugar transporter* |
| Traes_2AS_F55243E0C | 6,826,532-6,827,890 | F775_26609 | Putative nicotianamine synthase 2 | *Aegilops tauschii* | 97 | Nicotianamine synthase, S-adenosyl-L-methionine-dependent methyltransferase |
| Traes_2AS_66BFD95D4 | 6,832,418-6,834,861 | GSMUA_Achr11G19150_001 | Putative F-box domain containing protein | *Musa acuminata* | 23 | F-box domain, Protein of unknown function DUF295 |
| Traes_2AS_C8B6AF996 | 6,849,484-6,850,930 |  |  |  |  | P-loop containing nucleoside triphosphate hydrolase |
| Traes_2AS_150D52D59 | 6,864,263-6,866,588 | F775_19462 | GDSL esterase/lipase | *Aegilops tauschii* | 86 | Lipase, GDSL, SGNH hydrolase-type esterase domain |
| Traes_2AS_0CA29A19C | 6,882,712-6,895,168 | GRMZM2G154509 | Phosphoglycerate mutase-like protein isoform | *Zea mays* | 73 | Histidine phosphatase superfamily |
| Traes_2AS_86633353C | 6,897,466-6,901,759 | TRIUR3_13083 | Disease resistance protein RPM1 | *Triticum urartu* | 95 | Leucine-rich repeat domain, L domain-like, NB-ARC, P-loop containing nucleoside triphosphate hydrolase |
| Traes_2AS_DAA5EF278 | 6,926,483-6,930,173 | TRIUR3_03939 | Cytochrome P450 71C2 | *Triticum urartu* | 85 | Cytochrome P450 |
| Traes_2AS_600F98C3A | 6,959,604-6,963,872 |  | P-loop containing nucleoside triphosphate hydrolases superfamily protein |  |  | Adenylate kinase/UMP-CMP kinase, P-loop containing nucleoside triphosphate hydrolase |
| Traes_2AS_10B992D89 | 6,968,455-6,972,302 | TCM_001771 | 4-hydroxyphenylacetaldehyde oxime monooxygenase | *Theobroma cacao* | 51 | Cytochrome P450 |
| Traes_2AS_C1EA81EC4 | 7,039,670-7,043,193 |  | RuBisCO large subunit-binding protein subunit alpha, chloroplastic |  |  | Chaperonin Cpn60, GroEL-like apical domain |
| Traes_2AS_AD125CB18 | 7,055,379-7,058,871 | F775_52491 | arginase | *Aegilops tauschii* | 91 | Ureohydrolase |
| Traes_2AS_CFA978965 | 7,083,225-7,085,187 | F775_13313 | Beta-glucosidase 1 | *Aegilops tauschii* | 90 | Glycoside hydrolase superfamily |
| Traes_2AS_5233F6588 | 7,112,799-7,113,498 |  |  |  |  |  |
| Traes_2AS_6CE6AB560 | 7,118,296-7,123,325 | LOC100830175 | Putative disease resistance protein RGA3 | *Brachypodium distachyon* | 79 | Leucine-rich repeat domain, L domain-like, NB-ARC, P-loop containing nucleoside triphosphate hydrolase |
