# Supplementary figures and images for: Genome-wide association mapping for resistance to leaf rust, stripe rust and tan spot in wheat reveals potential candidate genes
Source: Theor Appl Genet. 2018 Mar 27;131(7):1405–22. doi: 10.1007/s00122-018-3086-6 (PMC6004277; doi:10.1007/s00122-018-3086-6)

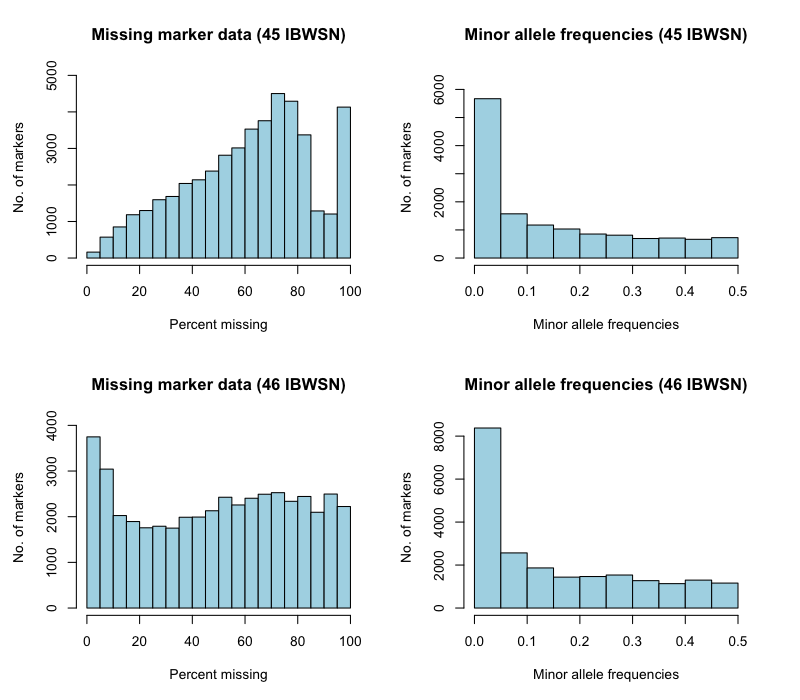

Supplement: Supplementary file 2 — Supplementary material 2 (TIFF 2189 kb) Supplementary Fig. 1: Distribution of missing data and minor allele frequency of markers in the 45th and 46th International Bread Wheat Screening Nursery entries [file 122_2018_3086_MOESM2_ESM.tiff]

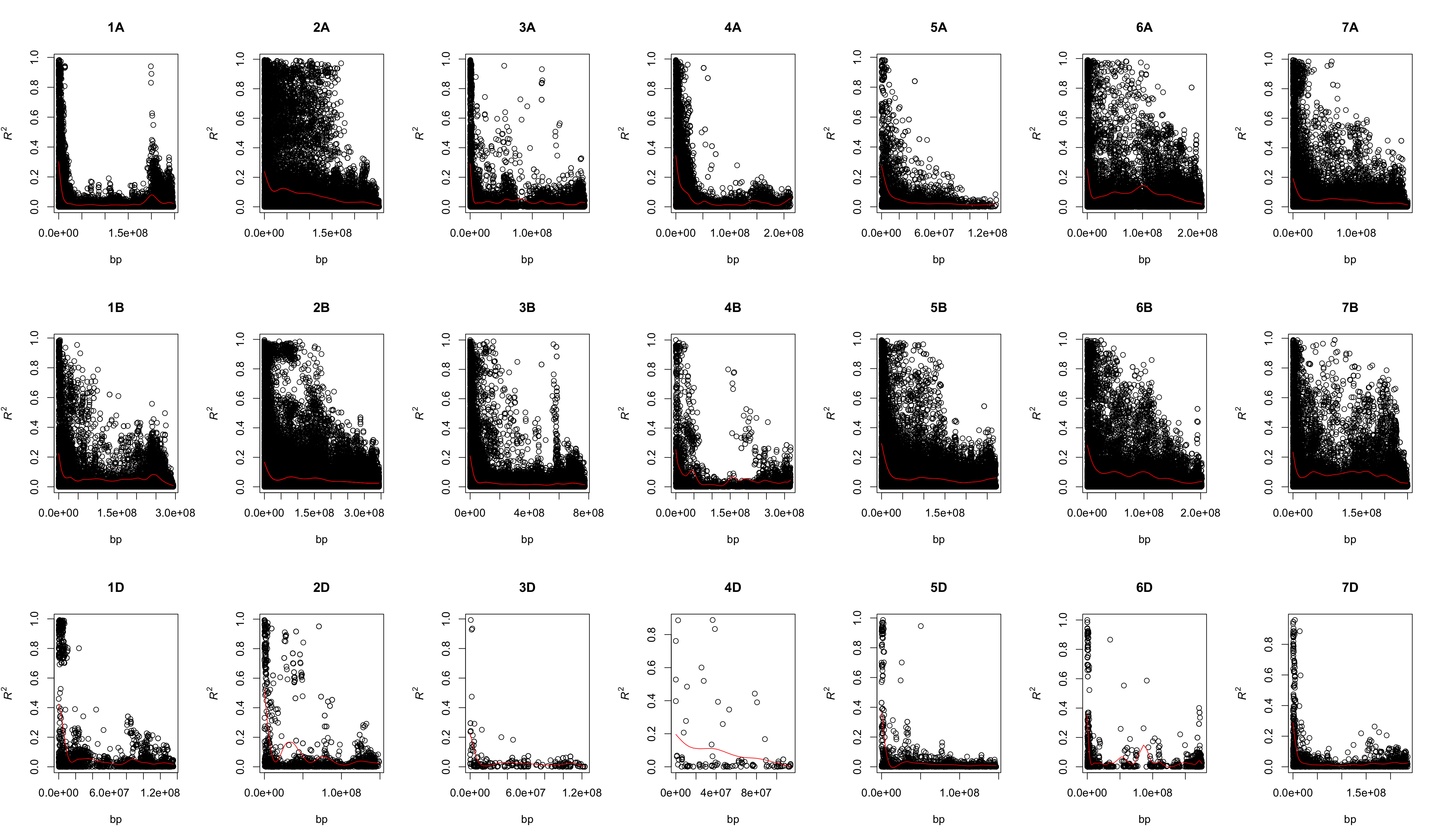

Supplement: Supplementary file 3 — Supplementary material 3 (TIFF 52505 kb) Supplementary Fig. 2: Scatter plot showing the linkage disequilibrium (LD) decay across the chromosomes. The physical distance in base pairs is plotted against the LD estimate (R2) for pairs of markers in the 45th International Bread Wheat Screening Nursery [file 122_2018_3086_MOESM3_ESM.tiff]

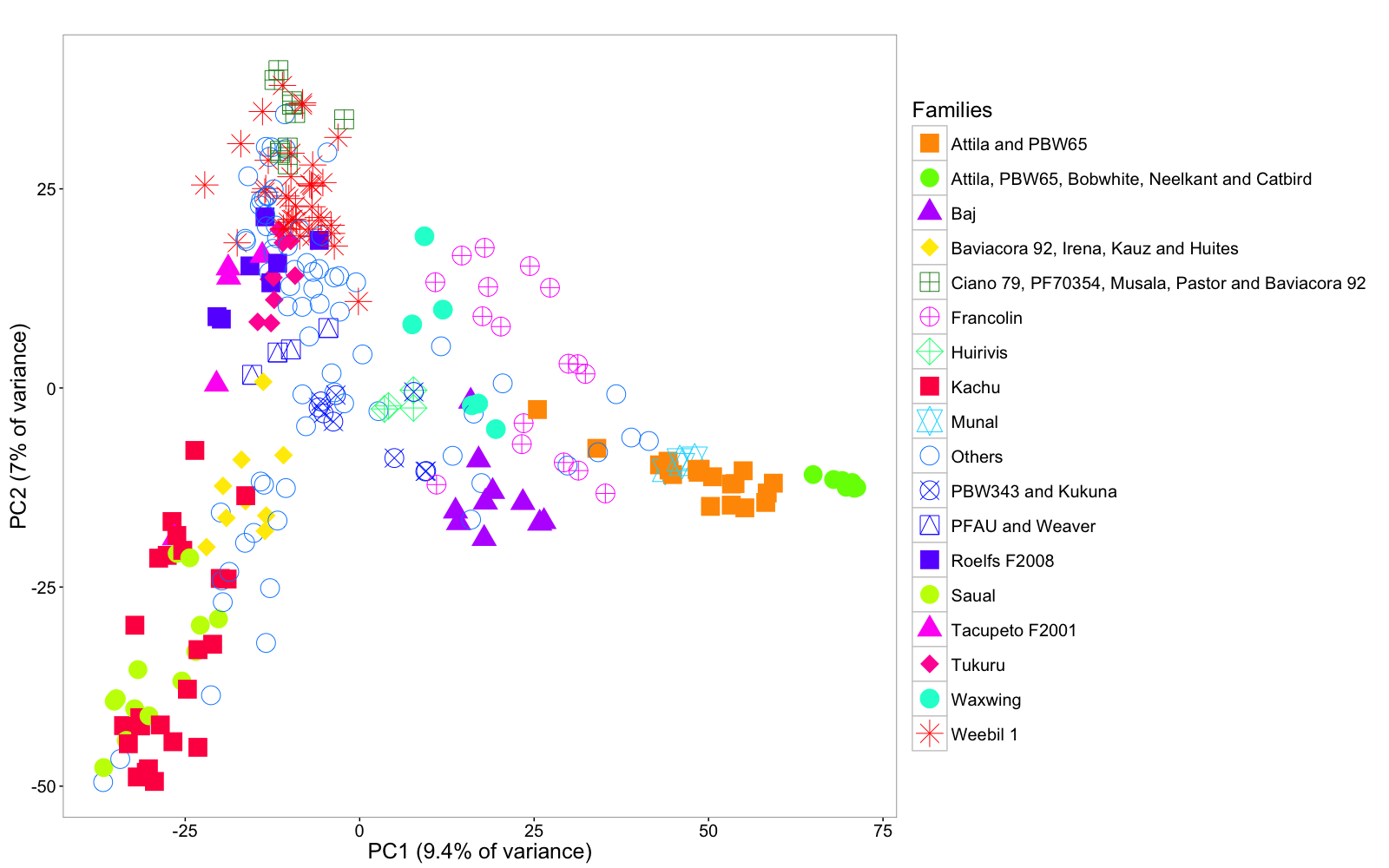

Supplement: Supplementary file 4 — Supplementary material 4 (TIFF 6252 kb) Supplementary Fig. 3: Principal component analysis and clustering of families in the 45th International Bread Wheat Screening Nursery [file 122_2018_3086_MOESM4_ESM.tiff]

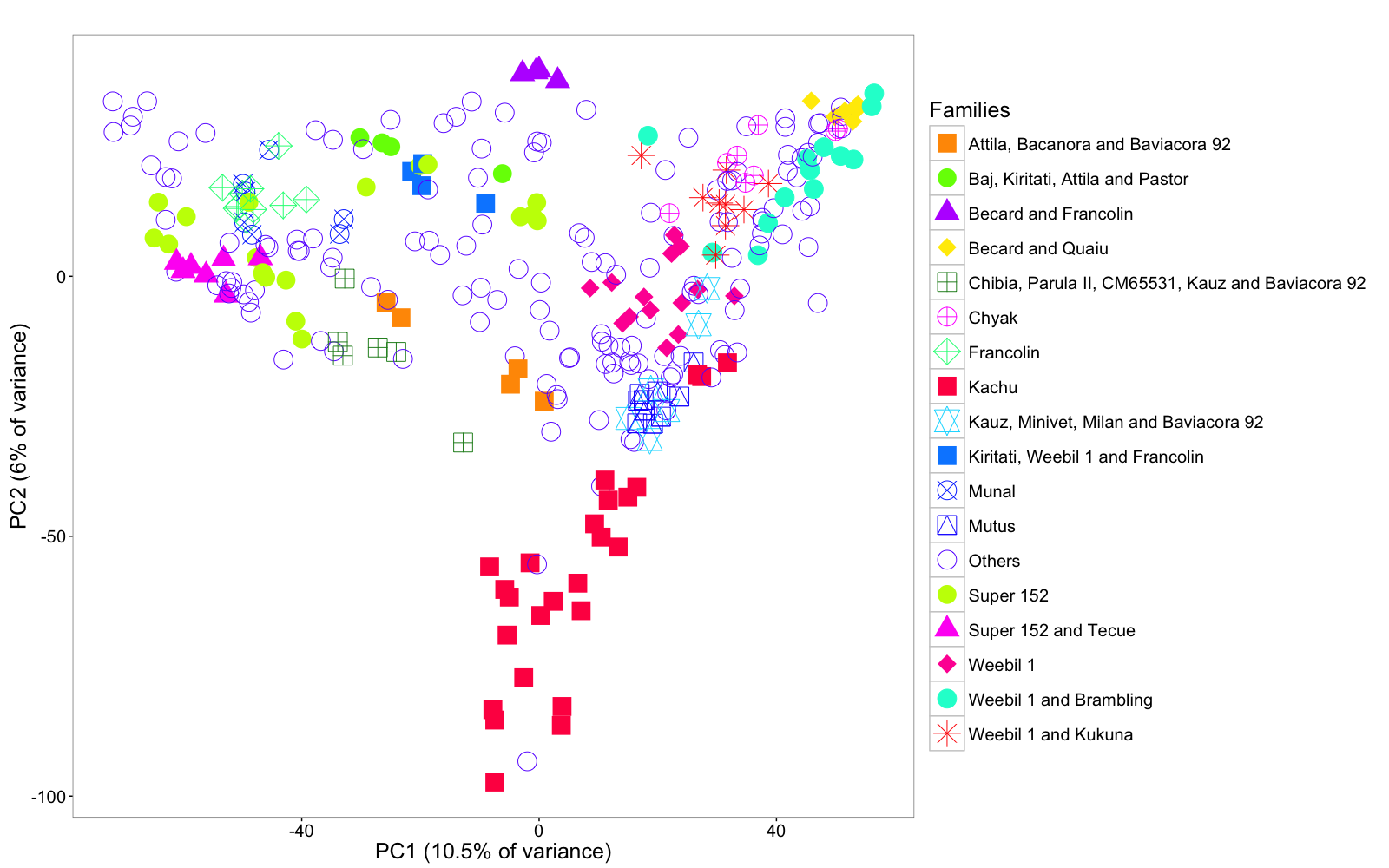

Supplement: Supplementary file 5 — Supplementary material 5 (TIFF 6252 kb) Supplementary Fig. 4: Principal component analysis and clustering of families in the 46th International Bread Wheat Screening Nursery [file 122_2018_3086_MOESM5_ESM.tiff]

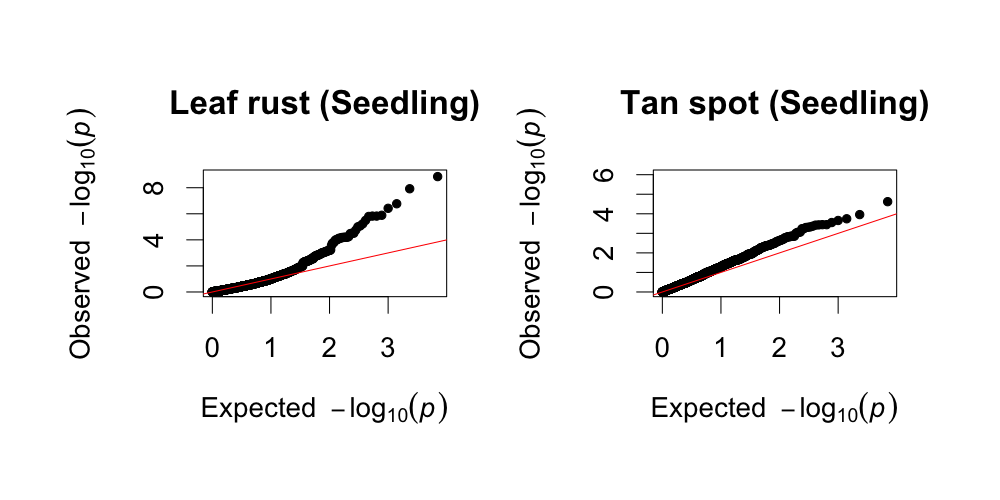

Supplement: Supplementary file 6 — Supplementary material 6 (TIFF 1955 kb) Supplementary Fig. 5: Quantile–quantile plots of p values comparing the uniform distribution of the expected –log10 p value to the observed –log10 p value for leaf rust and tan spot in the 45th International Bread Wheat Screening Nursery [file 122_2018_3086_MOESM6_ESM.tiff]

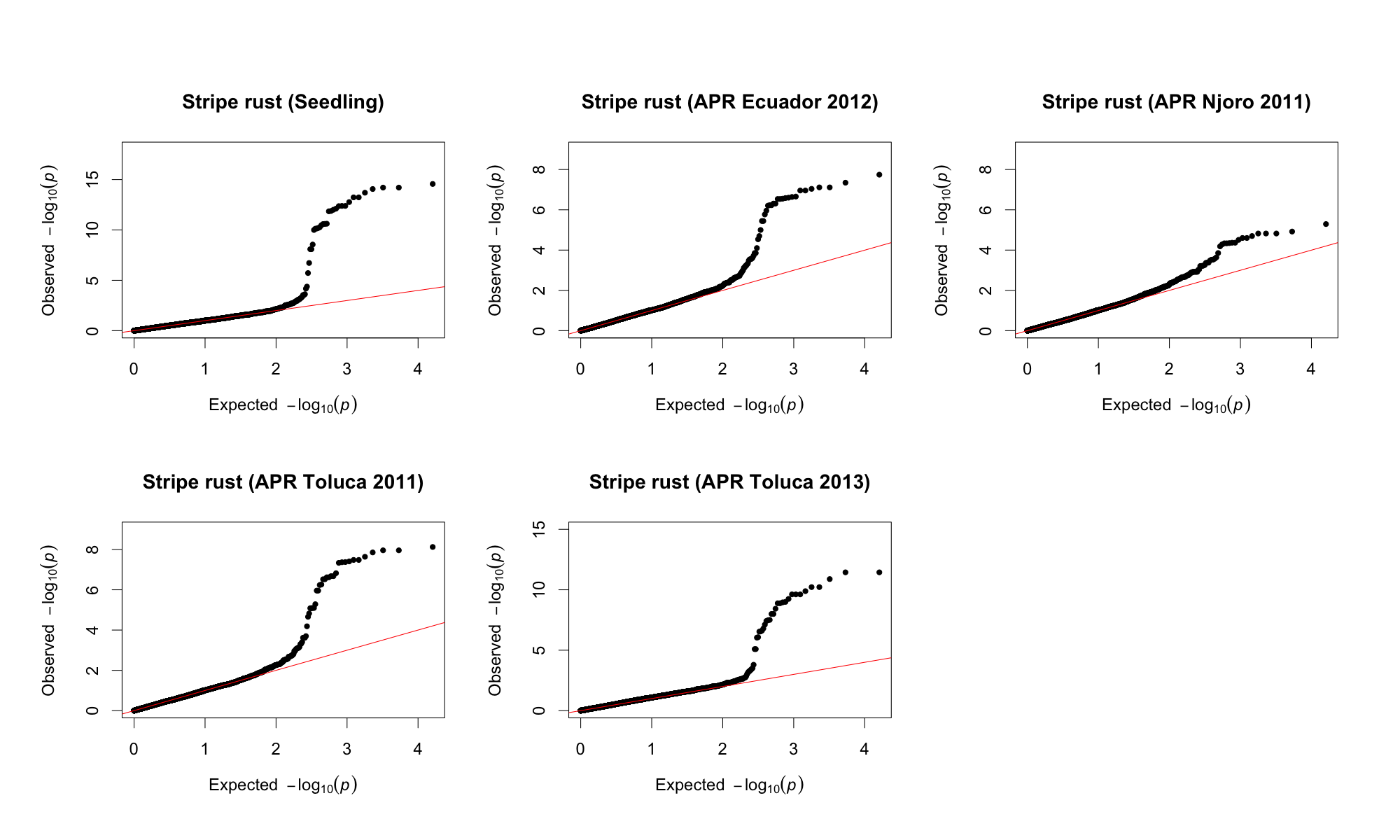

Supplement: Supplementary file 7 — Supplementary material 7 (TIFF 9377 kb) Supplementary Fig. 6: Quantile–quantile plots of p values comparing the uniform distribution of the expected –log10 p value to the observed –log10 p value for stripe rust seeding and adult plant resistance in the 46th International Bread Wheat Screening Nursery [file 122_2018_3086_MOESM7_ESM.tiff]

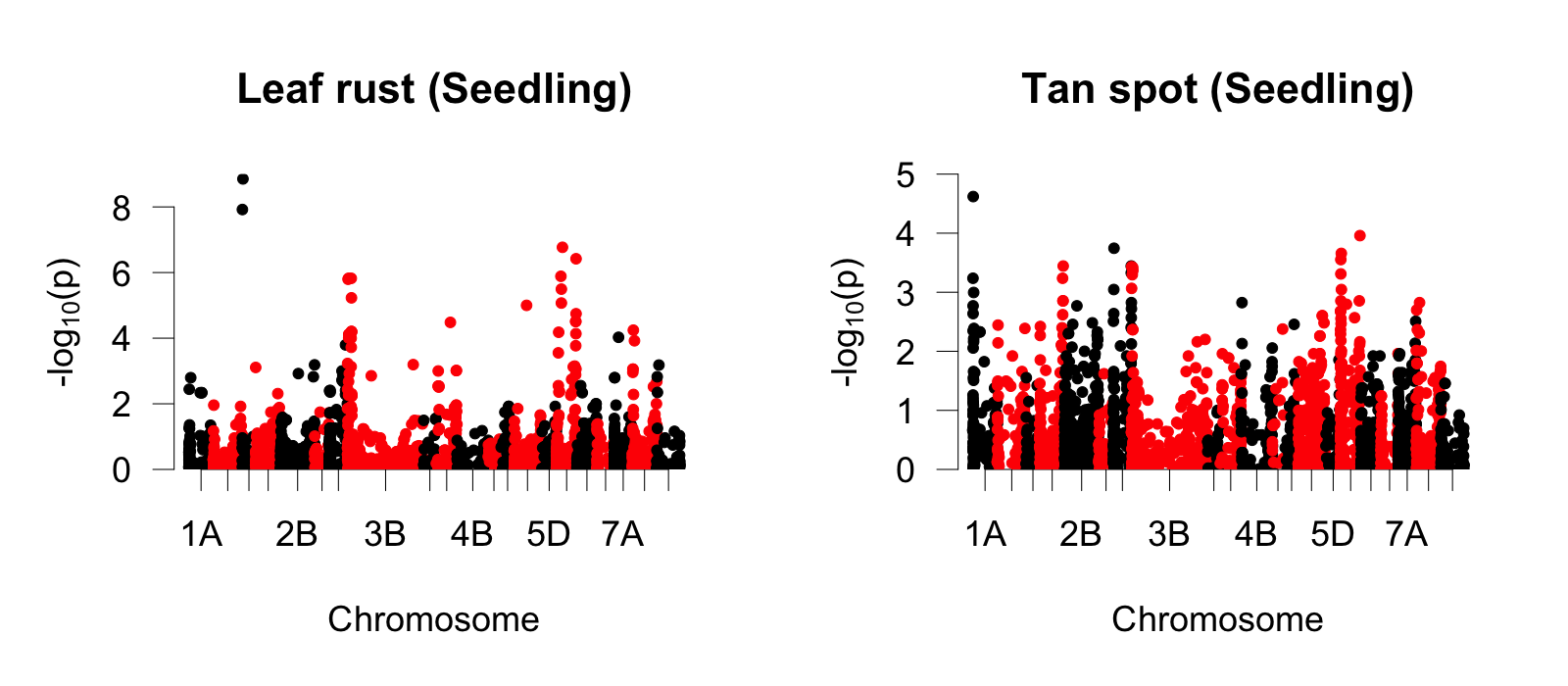

Supplement: Supplementary file 8 — Supplementary material 8 (TIFF 4377 kb) Supplementary Fig. 7: Manhattan plot showing –log10 p values of the markers for seedling resistance to leaf rust and tan spot in the 45th IBWSN [file 122_2018_3086_MOESM8_ESM.tiff]

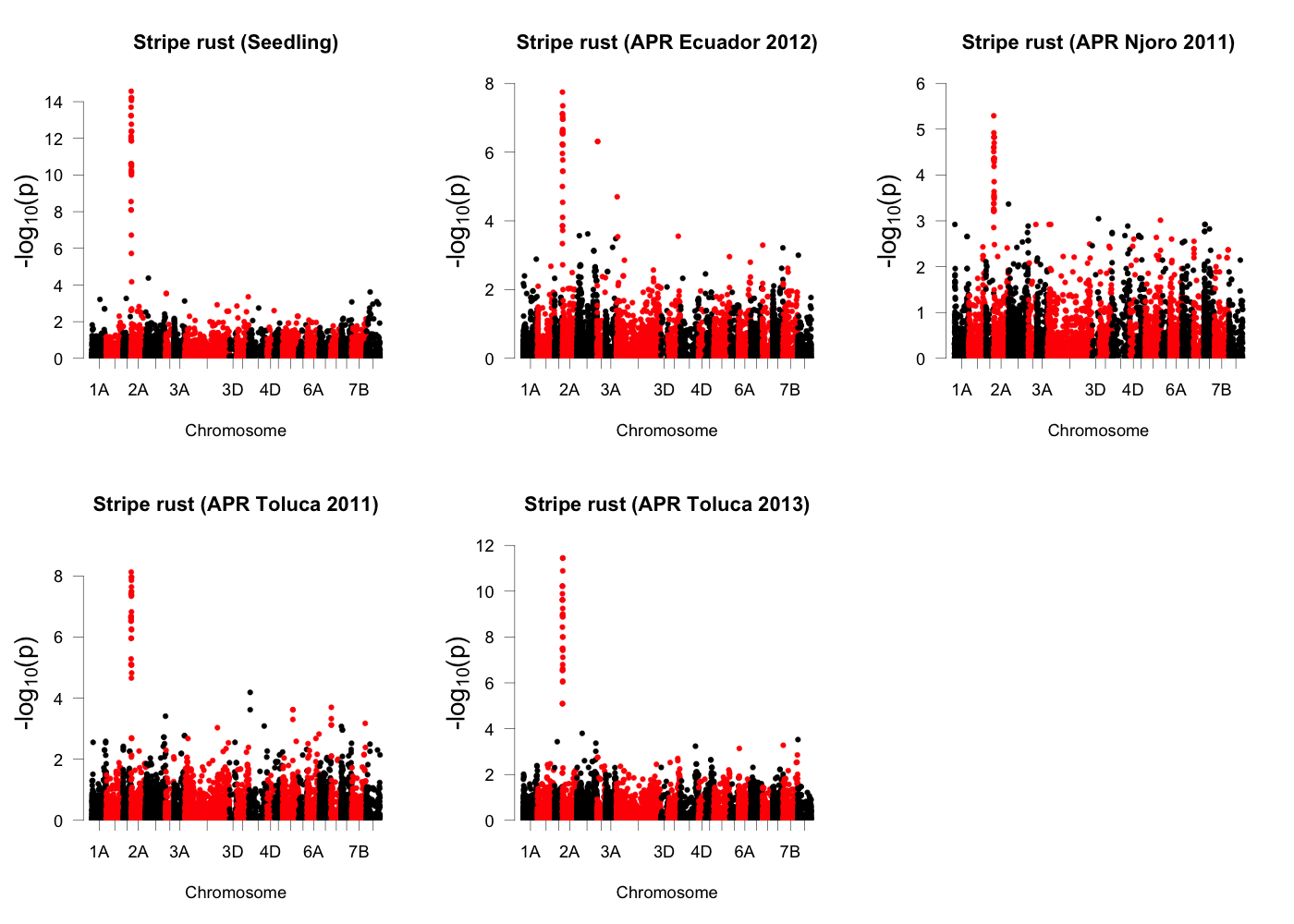

Supplement: Supplementary file 9 — Supplementary material 9 (TIFF 5471 kb) Supplementary Fig. 8: Manhattan plot showing –log10 p values of the markers for seedling and adult plant resistance to stripe rust in the 46th IBWSN [file 122_2018_3086_MOESM9_ESM.tiff]

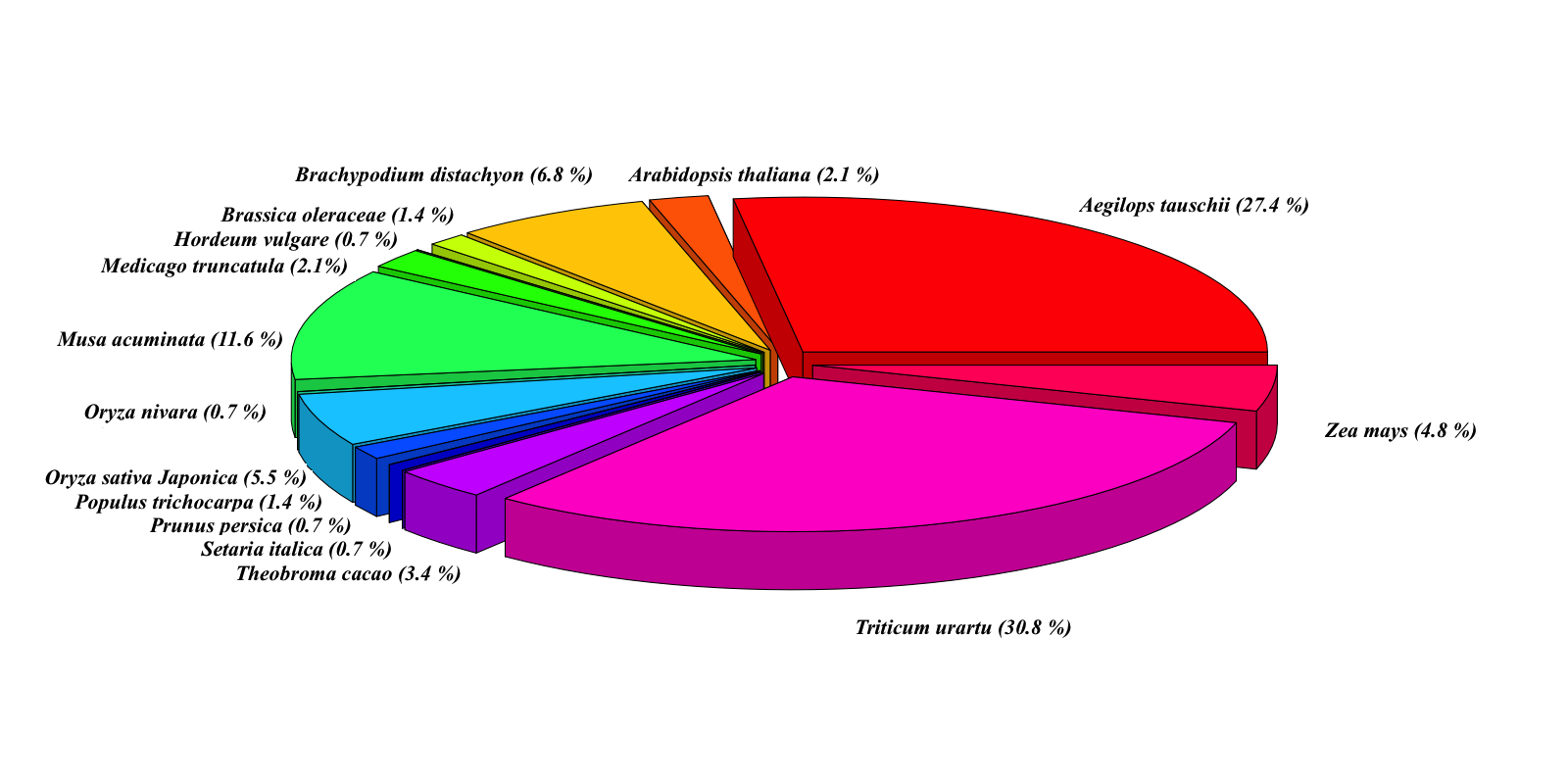

Supplement: Supplementary file 10 — Supplementary material 10 (TIFF 5002 kb) Supplementary Fig. 9: Pie chart showing the species with orthologs of highest similarity to the T. aestivum genes in a segment on chromosome 2AS [file 122_2018_3086_MOESM10_ESM.tiff]
